# Efficacy and Safety of Cellular Immunotherapy by Local Infusion for Liver Tumor: A Systematic Review and Meta-Analysis

**DOI:** 10.3389/fonc.2022.772509

**Published:** 2022-02-28

**Authors:** Shanshan Chen, Hualei Chen, Yongchao Zhang, Wei Li

**Affiliations:** Cancer Center, Beijing Ditan Hospital, Capital Medical University, Beijing, China

**Keywords:** liver neoplasms, cellular immunotherapy, local infusion, efficacy, safety, systematic review

## Abstract

**Background:**

Cellular immunotherapy has become a new and promising treatment for patients with liver tumor. However, as most immune cells are delivered by intravenous injection, the effect is limited and is likely to produce systemic toxicity. Here, the objective was to investigate the efficacy and safety of cellular immunotherapy by local infusion, which seems to be a promising approach and has not been well-studied.

**Methods:**

The PubMed, Web of Science, Embase, and Cochrane Library databases were searched to obtain literature. The overall response rate (ORR), overall survival (OS) rates, and adverse events were investigated to evaluate the effectiveness and safety of locoregional therapy. The methodological quality of the articles was assessed using the methodological index for non-randomized studies (MINORS) score. The meta-analysis was performed using Stata 15.0.

**Results:**

The eligible 17 studies involved a total of 318 patients. The random-effects model demonstrated that the ORR of local cell infusion therapy was 48% (95% confidence interval [CI]: 26%–70%). The pooled OS rate was 94% (95% CI: 83%–100%) at 6 months, 87% (95% CI: 74%–96%) at 12 months, and 42% (95% CI: 16%–70%) at 24 months. Subgroup analyses suggested that minimally invasive treatment and absence of metastasis were significantly associated with better ORR. Fourteen studies reported a variety of adverse events related to cell therapy by local perfusion. The most common complications after regional infusion of immune cells were myelosuppression (66%), fever (50%), gastrointestinal toxicity (22%), hepatic dysfunction (15%), and pleural effusion and/or ascites (14%).

**Conclusions:**

Immune cell therapy through local perfusion is effective for patients with liver cancer, with manageable toxicity. It demonstrates better prognosis when combined with minimally invasive therapy. Considering the potential limitations, more randomized controlled trials are needed to provide solid evidence for our findings.

## Introduction

Liver cancer, including primary liver cancer and liver metastasis, ranks sixth among the most common cancers in the world, and is recognized as the fourth leading cause of cancer-related death (1). Progression and recurrence after treatment are the principal reasons for the poor prognosis of patients with hepatocellular carcinoma (HCC), which accounts for 75%–85% of primary liver cancer. Meanwhile, the liver has been identified as a common site of metastasis, and the presence of liver metastasis is usually associated with lower survival rates (2). As patients are often diagnosed with liver cancer in the advanced stages, only limited treatment options are available, and the outcome fails to live up to expectations in most cases (3). On one hand, surgery, ablation, and arterial embolization are therapies with a locoregional scope of targeting lesions, which probably leads to local changes in tumor biology and contributes to postoperative recurrence to some extent (4, 5). On the other hand, chemotherapy and radiotherapy easily bring about resistance through different mechanisms (6–9). In addition, the epidemiology of hepatocellular carcinoma is gradually changing over the past dozen years. For example, HCC patients are older and metabolic disorders have become a growing cause of liver cancer (10). Changes in etiology and clinical features complicates the management of liver cancer. Conventional treatments are not sufficient to satisfy the demands of individualized cancer therapy. Accordingly, there is an urgent need to develop new methods to aid liver cancer treatment and enhance the survival benefit for patients.

With the development of molecular biology and tumor immunology, cellular immunotherapy, a new and promising therapeutic strategy, has been promoted in recent years. Immune cells are isolated from blood and cultured *in vitro*. Based on the principles of immunology, these processed immune cells with antitumor properties can be injected into patients to kill tumor cells directly or indirectly. Previous studies have mainly involved lymphokine-activated killer (LAK) cells, cytokine-induced killer (CIK) cells, natural killer (NK) cells, dendritic cells (DC), tumor-infiltrating lymphocytes (TIL), chimeric antigen receptor (CAR)-engineered T (CAR-T) cells, CAR-engineered NK (CAR-NK) cells, and T cell receptor (TCR)-engineered T (TCR-T) cells. LAK cells and CIK cells are activated lymphocytes with direct killing effect. NK cells are major part of the innate immune response against viruses and tumors. By presenting antigens, DCs induce innate and adaptive immune responses. TILs are isolated and expanded ex vivo from lymphocytes that have infiltrated tumors. CAR and TCR gene engineered immune cells can specifically recognize tumor antigens by gene modifications. Cellular immunotherapy can strengthen the immune state of the body, alter the tumor immune microenvironment, and provide potential assistance in improving the therapeutic effect (11). Several studies and meta-analyses have shown that the combination of cellular immunotherapy and conventional therapy can have a longer-term and more stable anti-tumor effect (12, 13). However, most of the studies involve intravenous therapy, with limited efficacy and a tendency to generate systemic toxicity.

In this regard, some experts have proposed the idea of local infusion and have made relevant attempts. An animal experiment showed that a high concentration of LAK cells can be obtained in the first capillary bed 2 hours after local infusion, prompting the idea that local delivery of LAK cells may be more effective for tumor (14). This conclusion holds true for other cell therapy. The route of cell administration is an important factor for determining the biological distribution pattern of these cells, so it must be considered in the study of cellular immunotherapy. The typical methods for local infusion include transarterial therapies, such as transcatheter arterial embolization (TAE), hepatic arterial infusion chemotherapy (HAIC), transarterial chemoembolization (TACE), and transarterial radioembolization (TARE). As mature methods for locoregional treatment, they can selectively deliver drugs or radiation to the tumor site (15). The same should be true of immune cells. Theoretically, local infusion of immune cells can compensate for the disadvantage of the poor specificity of tumor antigens and avoid the systemic toxicity associated with intravenous infusion. However, there has been no systematic analysis of the efficacy and safety of cellular immunotherapy by local transfusion. The application value of immunocyte therapy with regional perfusion, such as *via* the hepatic artery or portal vein, remains controversial.

Therefore, we have summarized various cellular immunotherapy trials and have systematically evaluated the efficacy and safety of local perfusion with immune cells. We look forward to providing an objective foundation and meaningful reference for the clinical application of this treatment.

## Methods

This systematic review and meta-analysis has been carried out according to Preferred Reporting Items for Systematic Reviews and Meta-Analyses (PRISMA) guidelines (16).

### Search Strategy

PubMed, Web of Science, Embase and the Cochrane Library were searched comprehensively to obtain the literatures related to cellular immunotherapy by local infusion for liver tumor. The retrieval was performed by two researchers respectively and disagreements were resolved by a third expert. Subject words and free terms were combined for searching. There was a subtle tweak on retrieval language towards different databases. The search period was set dating from January 1, 1990 to March 8, 2021. Taking PubMed as an example, the specific retrieval strategy has been shown in **Table 1**. The detailed search strategies for other databases are presented in **Supplementary Methods**. Only the published literatures were included. If necessary, authors would be contacted for additional information.

**Table 1:** Retrieval strategy on PubMed.

Retrieval strategy	((lymphokine activated killer) OR (cytokine induced killer) OR (natural killer) OR (dendritic cell) OR (tumor infiltrating lymphocyte) OR (chimeric antigen receptor) OR (T cell receptor) OR LAK OR CIK OR NK OR DC OR TIL OR CAR-T OR CAR-NK OR TCR-T OR immunocytotherapy) AND (local OR regional OR hepatic OR HAI OR TACE OR (intraarterial OR intraartery OR intralesional OR intratumoral OR arterial OR artery)) AND (infusion OR perfusion OR transfusion OR implantation OR injection OR refusion OR reinfusion) AND (((hepatocellular OR hepatic OR liver OR hepatocyte*) AND (carcinom* OR cancer* OR neoplas* OR malign* OR tumor* OR metastas*)) OR HCC OR “Carcinoma, Hepatocellular”[Mesh] OR “Liver Neoplasms”[Mesh])

LAK, lymphokine-activated killer cells; CIK, cytokine-induced killer cells; NK, natural killer cells; DC, dendritic cells; TIL, tumor infiltrating lymphocytes; CAR-T, chimeric antigen receptor-engineered T cells; CAR-NK, chimeric antigen receptor-engineered NK cells; TCR-T, T-cell receptor-engineered T cells; HAI, hepatic arterial infusion; TACE, transarterial chemoembolization; HCC, hepatocellular carcinoma.

*, a wild-card term used to search for terms with different endings.

### Inclusion and Exclusion Criteria

The studies included in the review should meet the following criteria (1): patients were diagnosed with liver tumor (including primary liver cancer and liver metastasis) (2); immune cells applied included lymphokine-activated killer (LAK) cells, cytokine-induced killer (CIK) cells, natural killer (NK) cells, dendritic cells (DC), tumor-infiltrating lymphocytes (TIL), chimeric antigen receptor (CAR)-engineered T (CAR-T) cells, CAR-engineered NK (CAR-NK) cells, T-cell receptor (TCR)-engineered T (TCR-T) cells, and so on (3); immunocytotherapy was conducted *via* local infusion (hepatic artery, portal vein, or intratumoral injection) (4); at least one of the following outcomes should be provided, including overall response rate (ORR), overall survival (OS) rates, and adverse events. Age and gender of the patients and concurrent combination therapy were not limited.

The exclusion criteria are as follows (1): duplicate articles or data (2); irrelevant studies, animal studies, case reports, reviews, and conference papers (3); immune cells were injected only through systemic intravenous or intradermal infusion (4); efficacy and safety were not mentioned (5); number of cases ≤ 8 (6); researches with incomplete or missing data (7); language barriers.

### Data Extraction

Two authors independently performed data extraction. With title and abstract browsed, the literatures were preliminarily screened in compliance with the inclusion and exclusion criteria. Obviously unrelated articles would be excluded. Then the second screening was carried out by reading the full text to determine whether it was finally eligible. The extracted information included first author, published year, country, study design, number, age and gender of participants, neoplasm types, classes of cells injected, location of infusion, combination therapy, frequency of cell infusion, duration of treatment, follow up time, and outcomes. The primary outcome was effectiveness. Response rate and survival rate were used to evaluate the efficacy and overall response rate (ORR) was defined as sum of complete and partial response in this study. The secondary outcomes was safety, consisted of types of adverse events and their rates, as well as treatment-related mortality.

### Quality Evaluation

The methodological index for non-randomized studies (MINORS) score was used by two authors independently to evaluated the methodological quality of the articles (17). The MINORS tool is composed of eight items for non-comparative studies and four additional items for comparative studies. Items are scored as 0 (not reported), 1 (reported but inadequate) or 2 (reported and adequate). The version with a total score of 16 was provided for non-comparative studies while 24 for comparative studies. In this review, ≤8 was considered poor quality, 9−12 moderate quality, and ≥13 good quality for non-comparative studies. For comparative studies, ≤12 was considered poor quality, 13−18 moderate quality, and ≥19 good quality. Items 1 to 12 are as follows (1): a clearly stated aim (2); inclusion of consecutive patients (3); prospective collection of data (4); endpoints appropriate to the aim of the study (5); unbiased assessment of the study endpoint (6); follow-up period appropriate to the aim of the study (7); loss to follow up less than 5% (8); prospective calculation of the study size (9); an adequate control group (10); contemporary groups (11); baseline equivalence of groups (12); adequate statistical analyses.

### Statistical Analysis

Stata 15.0 was used for meta-analysis of single group rate. The pooled effect size (ES) and 95% confidence interval (CI) were constructed using fixed-effect or random-effects meta-analysis. After data processing, the heterogeneity test was carried out by Q test and *I^2^* statistic. If *I^2^* < 25%, it was judged that the heterogeneity was small; if 25% < *I^2^* < 50%, it was judged moderate heterogeneity; if *I^2^* > 50%, then substantial heterogeneity. When there was statistical homogeneity among the studies (*P* > 0.10, *I^2^* < 25%), the fixed-effect model was selected for analysis; when there was statistical heterogeneity among the studies (*P* < 0.10, *I^2^* > 25%), the random-effects model was used for analysis. If sufficient data were available, we performed subgroup analyses. Sensitivity analysis was performed to evaluate the influences of individual studies on the pooled effect sizes. Funnel plot and Egger’s test were used to examine publication bias. *P* < 0.05 indicated that the results were of statistical significance.

## Results

### Study Selection and Search Results

**Figure 1:**
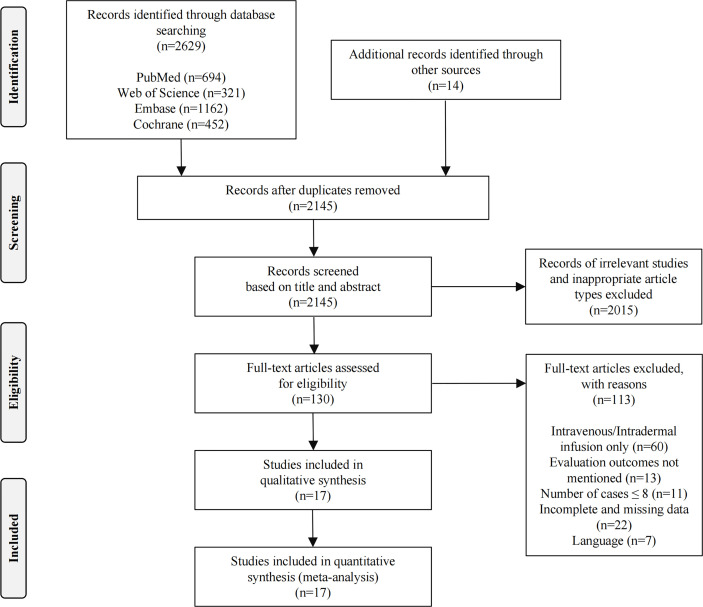
Flow diagram of search results according to PRISMA.

Initially, 2643 related studies were retrieved (694 articles in PubMed, 321 articles in Web of Science, 1162 articles in Embase, 452 articles in the Cochrane Library, and 14 articles from other sources). After eliminating 498 duplicate studies, a total of 2145 articles remained. Title and abstract screening resulted in the exclusion of 1880 unrelated studies, 93 animal studies, 11 case reports, 22 reviews, and nine conference papers, leaving 130 articles for full-text analysis. Through full-text review, we removed 113 studies: 60 were not in accordance with the infusion method studied, 13 had no evaluation endpoints, 11 had ≤8 patients, 22 had incomplete or missing data, and 7 had language barriers. Ultimately, 17 articles were considered eligible for further study (18−34). **Figure 1** presents the flow diagram of the search results according to PRISMA guidelines.

### Characteristics of the Included Studies

The 17 studies included involved a total of 381 participants: 261 patients in 12 articles were definitely diagnosed with HCC and 59 patients in six articles were diagnosed with liver metastasis. There were five randomized controlled trials (RCTs), nine prospective studies, and three retrospective studies. Eight studies had been performed in China, six in Japan, and one each in Germany, Italy, and France. Seven studies used LAK cells, four used CIK cells, three used DC, one combined DC with CIK and γδ T cells, and one each utilized cytotoxic T lymphocytes (CTL) and NK cells. Further, immune cell treatment in nine studies had been combined with minimally invasive treatments such as TAE or radiofrequency ablation (RFA). The different geographic locations and combined therapies were possible sources of heterogeneity. **Table 2** shows the relevant information on the characteristics of the included studies.

**Table 2:** Characteristics of included studies.

Study	Country	Study design	Number of patients	Age (years)	Gender (male: female)	Neoplasm	Cell type	Location of infusion	Combination therapy	Frequency of cell infusion	Duration of treatment	Follow up time
Komatsu et al., 1990 (18)	Japan	Prospective	13	52 (36−71)	10: 3	9 HCC and 4 liver metastases	LAK	Hepatic artery	–	Once a week (One cycle)	2−15 cycles	NA
Aruga et al., 1991 (19)	Japan	Prospective	15	39−72	14: 1	13 HCC and 2 liver metastases	CTL	Hepatic artery	–	NA	1−5 times	6−25 months
An et al., 1994 (20)	China	Prospective	40	33−69	36: 4	Liver cancer	LAK	Vein and hepatic artery	TAI • TAE	8−9 times (Once per 3 days) through vein and 1−2 times through hepatic artery (One course)	1−2 courses	NA
Keilholz et al., 1994 (21)	Germany	Prospective	15	NA	NA	Liver metastases	LAK	Portal vein or hepatic artery	–	1−2 times (14 days for one course)	Courses repeated at 3-monthly intervals in the absence of disease progression	NA
Kawata et al., 1995 (22)	Japan	RCT	12	55.9 ± 11.0	10: 2	HCC	LAK	Hepatic artery	Chemotherapy	2−3 times during 3 weeks	NA	NA
Wang et al., 1996 (23)	China	Retrospective	14	40−64	14: 0	HCC	LAK	Portal vein and hepatic artery	Chemotherapy	3 times through hepatic artery and 2 times through portal vein (Once every other day)	NA	NA
Ferlazzo et al., 1997 (24)	Italy	Prospective	9	30−75	6: 3	1 HCC and 8 liver metastases	LAK	Tumor	–	A single weekly injection	Four cycles (All patients but one completed)	6−8 months
Xie et al., 2000 (25)	China	RCT	21	44.6 (34−70)	16: 5	Liver cancer	LAK	Hepatic artery	TACE	NA	NA	NA
Xu et al., 2004 (26)	China	Retrospective	36	NA	NA	HCC	CIK	Vein and tumor	TACE + PEI	Twice a week, 4−6 times through vein and 4−6 times into tumor (One cycle)	2 cycles	NA
Nakamoto et al., 2007 (27)	Japan	Prospective	10	45−79	9: 1	HCC	DC	Hepatic artery	TAE	NA	One time	NA
Shi et al., 2007 (28)	China	Retrospective	38	NA	NA	HCC	CIK	Hepatic artery and tumor	TACE	Twice a week, one time through hepatic artery and 4−6 times into tumor	NA	≥ 1 year
Cui et al., 2008 (29)	China	Prospective	21	53 (24−70)	8: 13	Liver metastases	CIK	Hepatic artery	Chemotherapy	Day 7, 14, 16 in one cycle (28 days)	2.2 ± 1.2 cycles	10 (3−21) months
Weng et al., 2008 (30)	China	RCT	45	43 (29−60)	31: 14	HCC	CIK	Hepatic artery	TACE + RFA	Fortnightly (Successive 4 times as a course)	8 or 10 infusions	18 months
Nakamoto et al., 2011 (31)	Japan	Prospective	13	68.2 ± 9.1	9: 4	HCC	OK432-DC	Hepatic artery	TAE	NA	One time	≥ 360 days
Xu et al., 2013 (32)	China	RCT	40	Median 51	33: 7	HCC	DC + CIK+ γδ T	Vein and tumor	TACE + PMCT	NA	NA	6−36 months
Adotevi et al., 2018 (33)	France	Prospective	9	60 (51−66)	6: 3	Liver metastases	NK	Hepatic artery	Cetuximab	NA	One time	NA
Kitahara et al., 2020 (34)	Japan	RCT	14	70.1 ± 7.1	9: 5	HCC	DC	Tumor	RFA	NA	One time	62 (30−105) months
			16	69.9 ± 7.4	9: 7	HCC	OK432-DC	Tumor	RFA			

RCT, randomized controlled trial; HCC, hepatocellular carcinoma; LAK, lymphokine-activated killer cells; CTL, cytotoxic T lymphocytes; CIK, cytokine-induced killer cells; DC, dendritic cells; γδ T, gamma delta T cells; NK, natural killer cells; TAI, transarterial infusion chemotherapy; TAE, transcatheter arterial embolization; TACE, transarterial chemoembolization; PEI, percutaneous ethanol injection; RFA, radiofrequency ablation; PMCT, percutaneous microwave coagulation therapy; NA, not available.

### Quality Assessment

**Figure 2:**
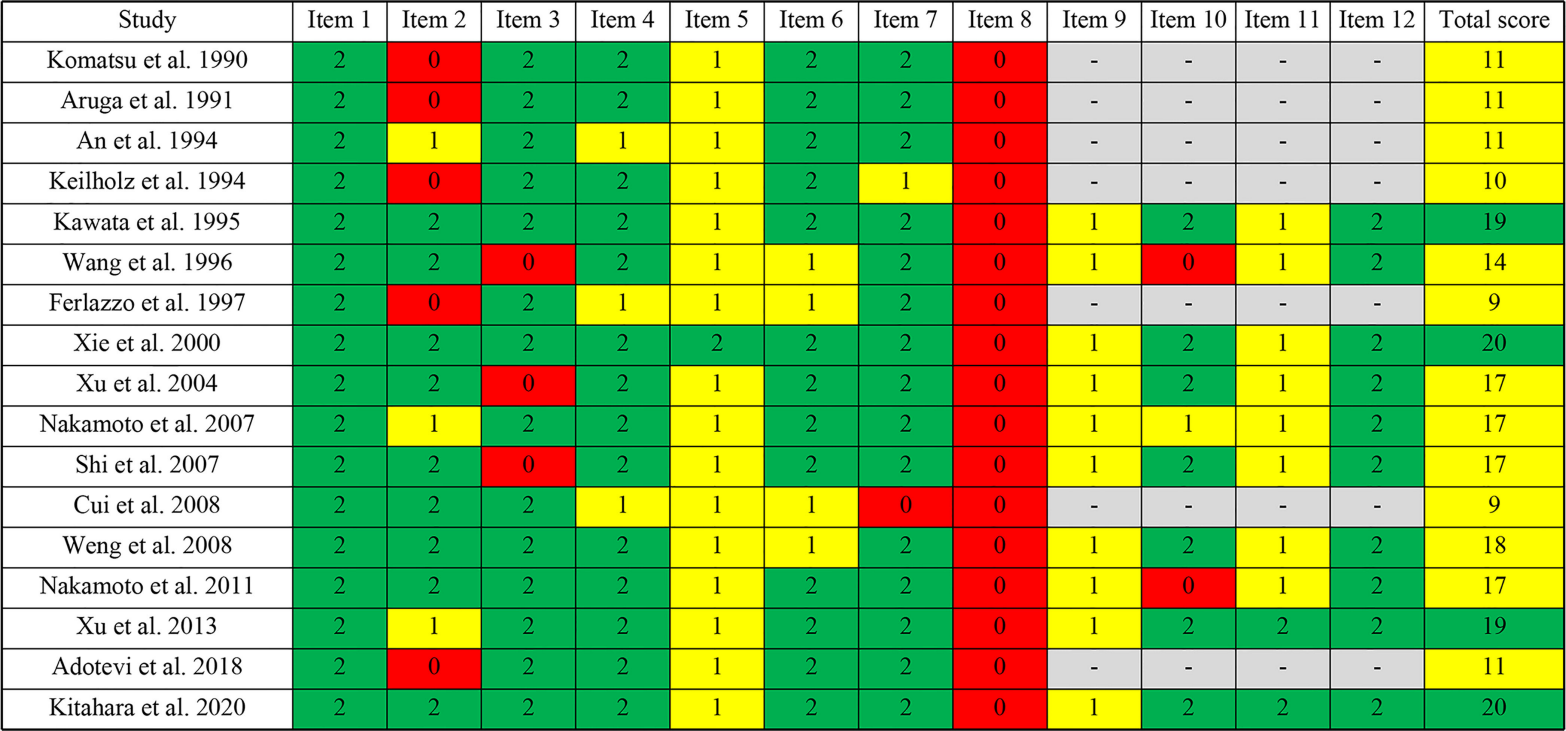
Risk of bias in each study assessed using the MINORS tool.

**Figure 3:**
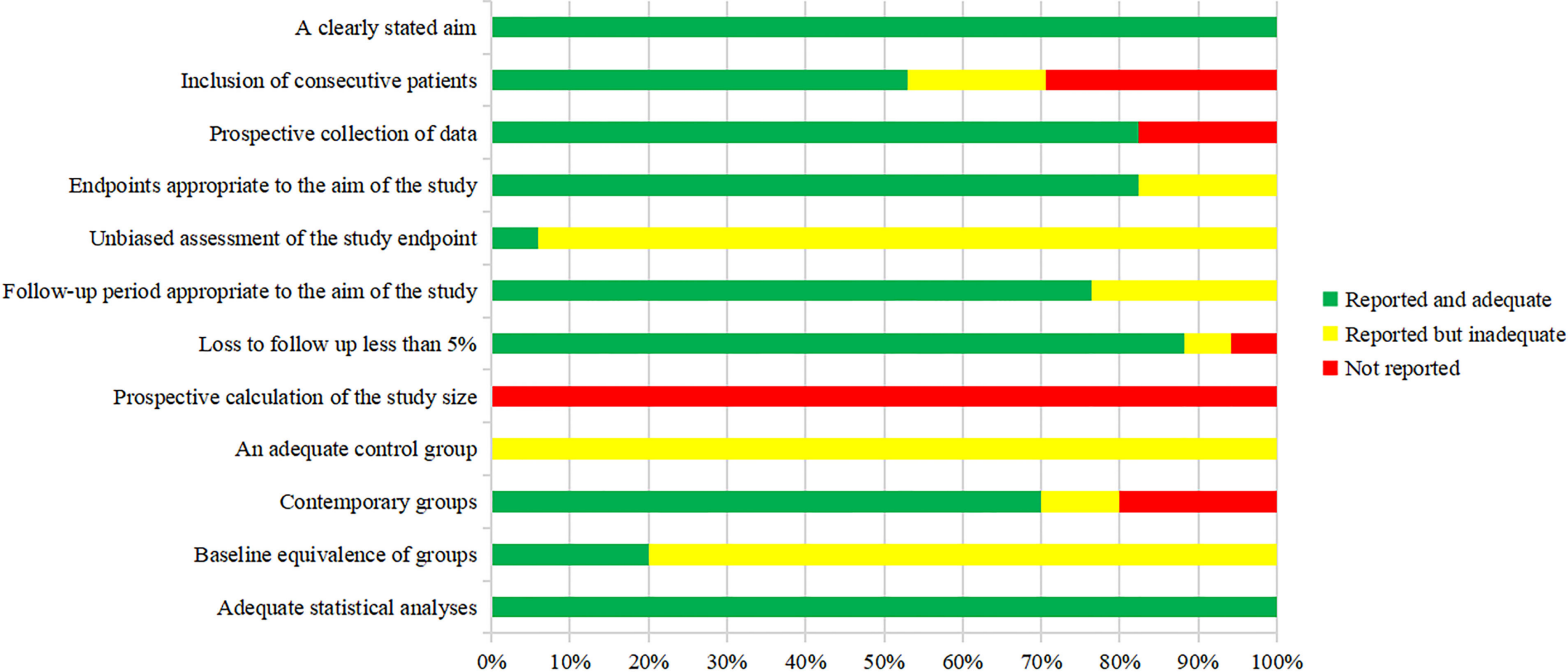
Risk of bias across studies assessed using the MINORS tool.

The methodological quality of the included studies was assessed by the MINORS tool. The scores are shown in **Figures 2** and **3**. Seven reports were non-comparative studies evaluated by eight items; the remaining 10 were comparative studies with an additional four criteria. Notably, no study had a prospective calculation of the study size. Based on separate grading standards, four studies had low risk of bias, with a total score indicating good quality; 13 studies were identified as moderate quality.

### Study Results

**Figure 4:**
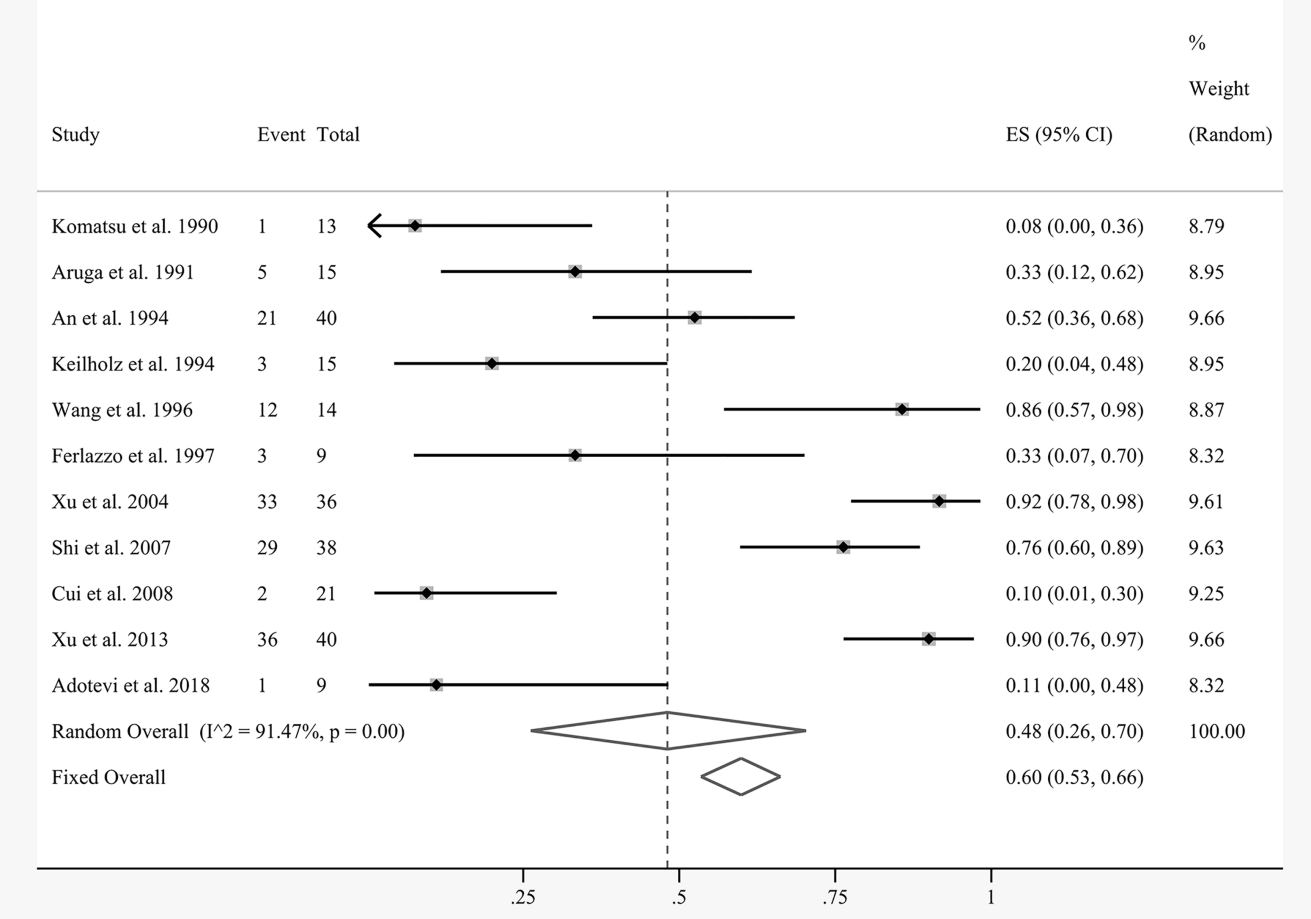
Forest plot showing pooled analysis of ORR.

**Figure 5:**
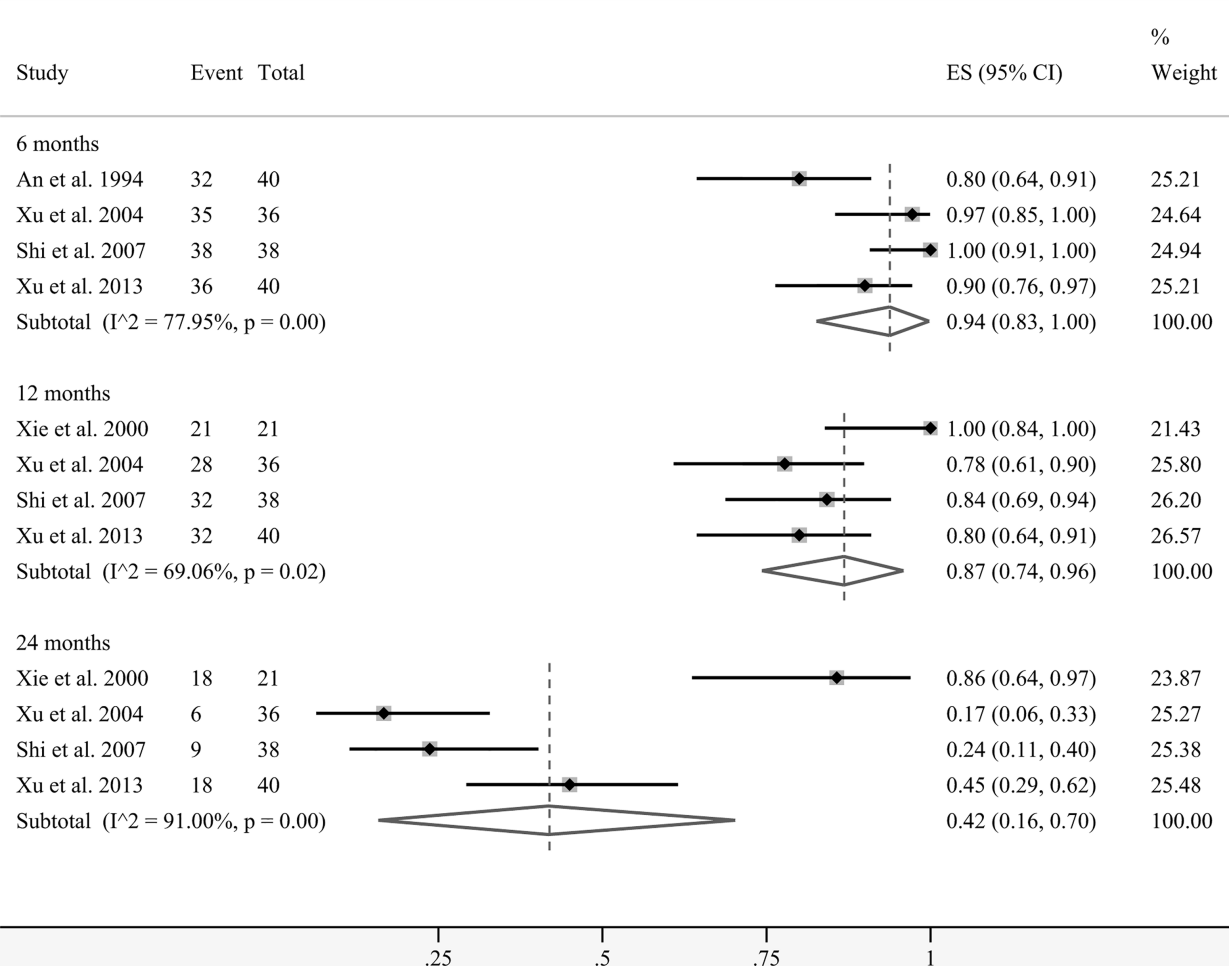
Forest plots showing pooled analyses of overall survival rates at 6, 12, and 24 months.

The efficacy results of the meta-analysis are shown in **Figures 4** and **5**. Due to the nature of the single-arm rate study, the corresponding outcome indices were pooled directly. Eleven articles were included for pooled analysis of the overall response rate (ORR), which equaled the complete response (CR) plus partial response (PR). There was substantial statistical heterogeneity between the involved studies (*I^2^* = 91.47%, *P* = 0.00). The random-effects model demonstrated that the pooled ORR of local cell infusion therapy was 48% (95% confidence interval [CI]: 26%–70%) (**Figure 4**).

A total of five studies presented data on the overall survival (OS) rates at several time points. Coincidentally, the patients in these groups all received interventional treatment as combined therapy. **Figure 5** demonstrates that the pooled survival rates of patients undergoing regional cellular immunotherapy in combination with transarterial therapy at 6, 12, and 24 months were 94% (95% CI: 83%–100%), 87% (95% CI: 74%–96%), and 42% (95% CI: 16%–70%), respectively.

Subgroup analyses were performed based on cell type, combination treatment, region, and primary or secondary cancer status. Subgroups with <3 studies after classification were not included in the analyses. **Table 3** shows that combination therapy with minimally invasive intervention seemed to be significantly associated with higher ORR compared with cell therapy alone (0.79 vs. 0.22, *P* = 0.014). Furthermore, the HCC group had a far greater ORR than the liver metastases group (0.87 vs. 0.13, *P* = 0.008). There was no statistical difference between the other two pairs (cell type, *P* = 0.442; region, *P* = 0.152). **Supplementary Figures 1**-**8** present the forest plots of each group. The meta-regression analysis suggested that interventional management and metastasis status might influence the effectiveness of local cellular immunotherapy.

**Table 3:** Subgroup analysis of ORR.

Possible sources of heterogeneity	Subgroup	Number of studies	Heterogeneity		ORR (95% CI)	*P* value
			*I^2^* (%)	*P* value		
Cell type	LAK	5	83.39	0.00	0.40 (0.15, 0.67)	0.442
	CIK	3	95.76	0.00	0.61 (0.14, 0.98)
Combination therapy	Cell therapy alone	4	6.43	0.36	0.22 (0.11, 0.35)	0.014
	Combined with minimally invasive treatment	4	85.3	0.00	0.79 (0.60, 0.94)
Region	Asia	8	92.49	0.00	0.58 (0.32, 0.81)	0.152
	Europe	3	0.00	0.57	0.21 (0.07, 0.37)
Metastasis status	HCC	4	21.75	0.28	0.87 (0.80, 0.92)	0.008
	Liver metastases	3	0.00	0.69	0.13 (0.04, 0.25)

ORR, overall response rate; LAK, lymphokine-activated killer cells; CIK, cytokine-induced killer cells; HCC, hepatocellular carcinoma.

**Figure 6:**
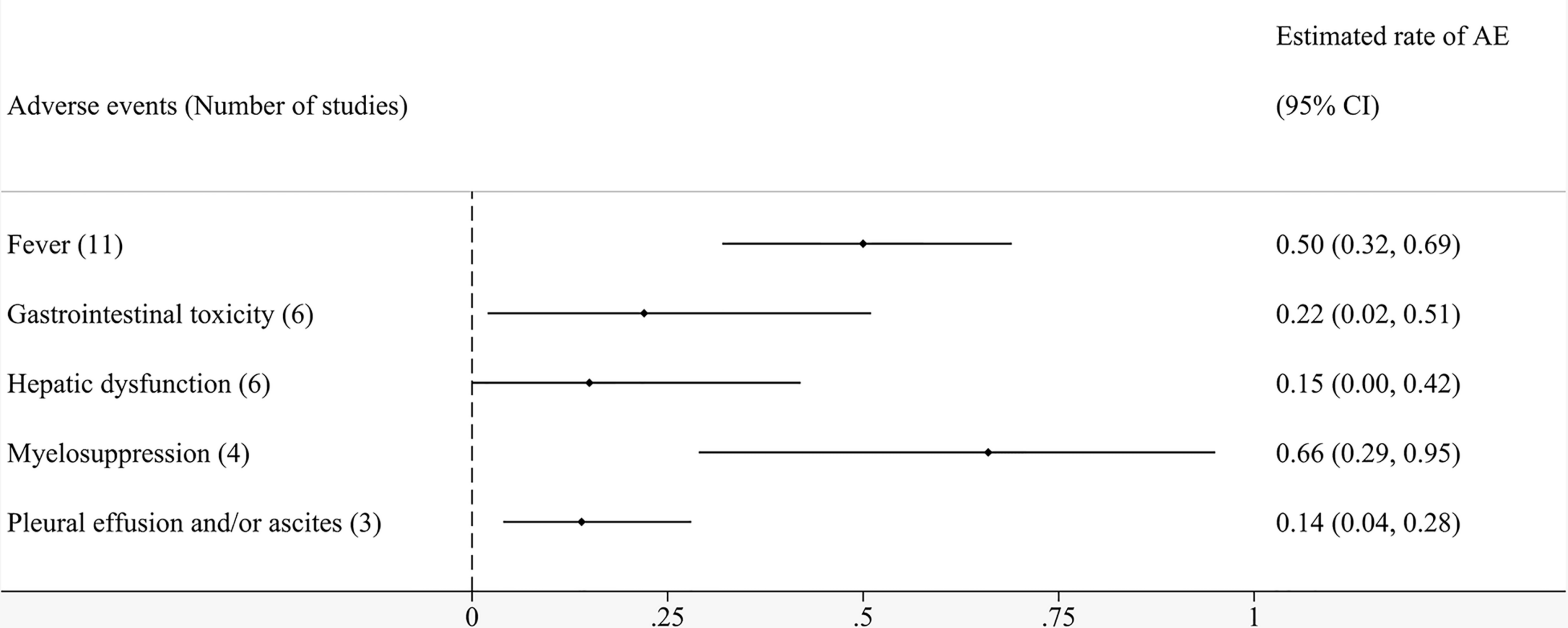
Pooled analyses on incidence of various adverse events.

Fourteen studies reported adverse events related to local perfusion cell therapy. **Table 4** shows that the complications patients were likely to experience after regional infusion of immune cells primarily included fever, gastrointestinal toxicity, hepatic dysfunction, myelosuppression, and pleural effusion and/or ascites. **Figure 6** shows the pooled analyses on the proportion adverse reactions in the patients. The estimated rates of fever, gastrointestinal toxicity, hepatic dysfunction, myelosuppression, and pleural effusion and/or ascites were 50% (95% CI: 32%–69%), 22% (95% CI: 2%–51%), 15% (95% CI: 0%–42%), 66% (95% CI: 29%–95%), and 14% (95% CI: 4%–28%), respectively (**Supplementary Figures 9**-**13**). Although reported in most of the studies, fever did not have the highest incidence. Myelosuppression was the most common adverse event because of its uppermost estimated rate. Symptomatic treatment relieved nearly all complications.

**Table 4:** Relevant adverse events.

Study	Number of patients	Cell type	Combination therapy	Adverse events					
				Fever	Gastrointestinal toxicity	Hepatic dysfunction	Myelosuppression	Pleural effusion and/or ascites	Other adverse events
Komatsu et al., (18)	13	LAK	–	13	2	1	NA	2	Dyspnea 1; headache 1; eosinophilia 4; hypotension 1
Aruga et al., (19)	15	CTL	–	6	NA	1	NA	1	NA
An et al., (20)	40	LAK	TAE	17	2	NA	12	NA	Gallbladder edema 3; alopecia 4; premature ventricular complexes 1; tachycardia 1
Keilholz et al., (21)	15	LAK	–	NA	5	Occurrence	NA	NA	NA
Kawata et al., (22)	12	LAK	–	11	NA	NA	NA	3	Renal insufficiency 1
Wang et al., (23)	14	LAK	Chemotherapy	2	12	0	12	NA	Urticaria 3; pain and weakness 2; dyspnea 1; alopecia 13
Xu et al., (26)	36	CIK	TACE + PEI	15	NA	NA	NA	NA	NA
Nakamoto et al., (27)	10	DC	TAE	5	2	NA	NA	NA	NA
Cui et al., (29)	21	CIK	Chemotherapy	NA	NA	9	8	NA	NA
Weng et al., (30)	45	CIK	TACE + RFA	11	NA	0	NA	NA	NA
Nakamoto et al., (31)	13	DC	TAE	12	0	NA	NA	NA	NA
Xu et al., (32)	40	DC + CIK + γδ T	TACE + PMCT	6	NA	NA	NA	NA	NA
Adotevi et al., (33)	9	NK	Cetuximab	NA	NA	7	9	NA	Febrile aplasia 2
Kitahara et al., (34)	30	DC	RFA	12	Nausea 1; abdominal pain 2	NA	NA	NA	NA

LAK, lymphokine-activated killer cells; CTL, cytotoxic T lymphocytes; CIK, cytokine-induced killer cells; DC, dendritic cells; γδ T, gamma delta T cells; NK, natural killer cells; TAE, transcatheter arterial embolization; TACE, transarterial chemoembolization; PEI, percutaneous ethanol injection; RFA, radiofrequency ablation; PMCT, percutaneous microwave coagulation therapy; NA, not available.

One study reported five deaths from hepatic failure, two from HCC rupture, two from varix rupture, one from subarachnoid hemorrhage, and one from respiratory failure. Another study reported one death from upper gastrointestinal bleeding. The other studies did not specify treatment-associated death.

### Sensitivity Analysis and Publication Bias

**Figure 7:**
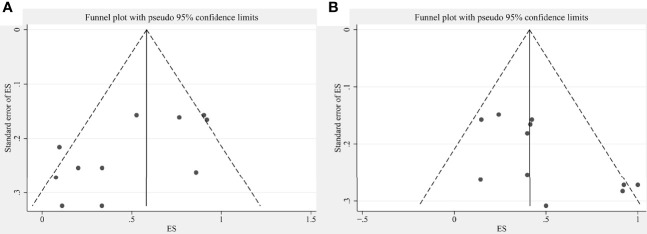
Funnel plots showing publication bias for **(A)** ORR pooled analysis and **(B)** fever rate pooled analysis.

This study conducted sensitivity analyses on the pooled effect sizes with articles >10. Results of sensitivity analysis were shown in **Supplementary Figure 14** (for pooled ORR) and **15** (for pooled fever rate). Changes in the two pooled effect sizes were not large, indicating that our results were stable. The publication bias was objectively evaluated with funnel plots and Egger’s test. The relative asymmetry of the funnel plots indicated the presence of publication bias, with *P* < 0.05 in Egger’s test (*P* = 0.025 for ORR pooled analysis; *P* = 0.035 for fever rate pooled analysis) (**Figure 7**). If there are <10 studies, the power of Egger’s test can be too low to distinguish chance from real asymmetry, so other pooled analyses were not considered.

## Discussion

Lacking typical symptoms in the early stage, liver cancer is one of the most common malignancies, with complicated therapeutic strategies. Transplantation, hepatectomy, ablation, transarterial embolization, chemotherapy, radiotherapy, and drug therapy can prolong survival to some extent. However, the curative effect remains poor, mainly owing to tumor recurrence and drug resistance of the liver cancer cells (35). By directly recognizing and killing tumor cells or enhancing the anti-tumor immune response, cellular immunotherapy has become a new method for improving the survival and curative effect in patients with liver tumor in recent years. Initial immunotherapy involves LAK cells, CIK cells, and other non-specific cell therapy, but the specificity is poor. In recent years, this problem has been solved with the development of specific cell therapy such as that involving CAR-T, CAR-NK, and TCR-T cells (11). However, most studies on cellular immunotherapy are still in phase I/II clinical trials. A meta-analysis showed that the administration of immune cells induced *in vitro* can decrease the early recurrence and mortality of postoperative HCC (36).

Cell-based immunotherapy has a strong anti-tumor effect on cancer cells. One of the treatments with the most potential in recent years, it has an extraordinary effect on blood tumors (37–39), but challenges remain, i.e., liver cancer, one of the solid tumors (40, 41). As most of the existing therapies use systemic infusion (i.e., intravenous infusion), these cells first reach the lung after entering the body, peak in 2–4 hours, and then are distributed to the liver, kidney, spleen, and other parts, tending to stabilize in 24 hours (42). Similar to the first-pass effect of drugs, the organ at which the infused immune cells first arrive is intercepted to the maximum extent. Therefore, for liver cancer, the cells cannot completely enter the tumor tissue. In contrast, direct regional infusion of immune cells can transport the cells effectively and avoid the physical barrier in systemic circulation (43). In theory, locoregional therapy can achieve the liver aggregation of immune cells to bring them into full effect. Different means of cell perfusion can influence the outcome of cancer therapy, and local injection improves the effectiveness and prolongs the response time (44, 45). Hence, there is more expectation that local infusion is used instead of systemic infusion alone. In recent reports on cell therapy, Schmidt et al. described the methods of combined antigen targeting, regional delivery, and improving the persistence of CAR-T cells in a malignant tumor microenvironment (40). Li et al. proved that CIK cell infusion therapy had a synergistic effect on the long-term survival of patients with liver cancer after minimally invasive treatment (12). However, no meta-analysis has focused on local perfusion in cell therapy. In the present review, we selected studies that transfused immunocytes *via* the hepatic artery, portal vein, or intratumoral injection instead of systemic infusion. It is worth noting that the sample size of each research was small and that the results varied greatly. There remains a lack of high-level evidence-based validation on the efficacy and safety of cellular immunotherapy *via* local perfusion.

Here, we comprehensively analyzed the results of 17 individual articles from four databases to investigate the efficacy and safety of local cell immunotherapy in the prognosis of patients with liver tumor. Meta-analysis demonstrated that the total response rate was 48% (95% CI: 26%–70%). The OS rates of patients treated with regional cellular immunotherapy in combination with interventional therapy at 6, 12, and 24 months were 94% (95% CI: 83%–100%), 87% (95% CI: 74%–96%), and 42% (95% CI: 16%–70%), respectively. The subgroup analysis and meta-regression results suggested that combination therapy and metastasis status might affect the outcome of immunocyte therapy. In particular, the ORR of local cell perfusion combined with interventional therapy was significantly higher than that of cell therapy alone. HCC had a superior response rate compared to liver metastasis, indicating that local cell immunotherapy has better application prospects in HCC. As the number of eligible studies in some subgroups was too small (n < 3) after classification, the pooled results and meta-regression analysis may be subject to bias. Therefore, we excluded groups with <3 studies from the subgroup analysis, such as the CTL group (n = 1), DC group (n = 1) and NK group (n = 1) in cell classification, and the chemotherapy group (n = 2) and cetuximab group (n = 1) in classification of combination therapy. We also excluded four studies due to their indeterminate cancer metastasis status. With respect to safety, one advantage of local cell infusion is that it has few special adverse effects. The most common adverse reactions were myelosuppression and fever, followed by gastrointestinal toxicity, hepatic dysfunction, and pleural effusion and/or ascites. These complications also frequently occur in routine treatment of liver cancer and can be well controlled by symptomatic treatment. No deaths directly related to local perfusion of cell therapy have been reported.

Immune cells are an important component of the tumor microenvironment (TME) and play critical roles in tumorigenesis (46). Tumor-antagonizing immune cells and tumor-promoting immune cells participate in tumor immunity regulation through producing various cytokines. Because of the multiple immunosuppressive mechanisms in the tumor microenvironment, immune cells in liver cancer often fail to work (47). It has been reported that CD4+CD25+Foxp3+ regulatory T cells (Tregs) play a immunosuppressive role in TME which contributes to HCC initiation and progression (48). CD3+, CD8+, activated NKs, and Foxp3+ T cells were also found to have a suppression function in HCC (49). Several included literatures demonstrated altered T cell subpopulations in patients after CIK cell infusions (26, 28, 30). In another included article, the level of CD4+CD25+FOXP3+/CD4+ cells in patients treated with cellular immunotherapy was significantly lower than before, suggesting that the immunosuppression status improved (32). We inferred that improving the composition of immune-cell subsets might be a crucial step for promoting tumor immunity in liver cancer. Cell therapy varies in function and efficacy, depending in part on the type of immune cells infused. It was a shame that only the LAK group and the CIK group were compared in the subgroup analysis, given the potential bias. The CIK group seemed to have a higher ORR than the LAK group, but the statistical difference was not significant (0.61 vs. 0.40, *P* = 0.442). CIK cells are a heterogeneous cell population comprised of CD3+CD56+ cells, CD3−CD56+ NK cells, and CD3+CD56− T cells, while LAK cells predominantly contain CD3−CD56+ NK cells and CD3+ T cells (50, 51). Some studies argued that CIK cells were better, as they showed easier availability and more effective cytotoxicity (52–54). In recent years, CAR and TCR gene engineered immune cells have gained increased attention due to their ability of specifically recognition. More large-scale studies are expected to optimize the strategy of cell therapy.

In view of the occult occurrence of liver neoplasms, patients are often in the middle and late stage when diagnosed, missing the chance for surgery. TACE is generally considered the first choice for these unresectable liver tumors. However, for most patients treated with TACE alone, even with repeated treatment, active cancer cells remain in the tumor lesions. It is difficult for patients with liver cancer to maintain long-term survival because the tumor cannot be completely eliminated by TACE. Applying local cell therapy as an auxiliary means to interventional therapy such as TACE to control localized advanced diseases is a novel and exciting prospect. Cell therapy after minimally invasive treatment in patients with liver cancer is helpful for removing tumor microlesions, preventing recurrence or metastasis, and ultimately improving the patient’s quality of life and prognosis (12, 55, 56). Immune cells injected into the tumor can significantly increase the number of working cells in the tumor area, and further kill the residual cancer cells after the interventional therapy. We have verified that our results are consistent with this viewpoint. The ORR and survival of combination groups were all elevated as compared with their respective control groups treated with interventional therapy alone (if any).

Our study has some limitations. There are differences in the patients’ disease severity, age, sex, and other basic characteristics, so there was large clinical heterogeneity between the studies. Only studies with commonly used immunocyte therapy were included, because normative nomenclature and classification of the therapy had not been provided. In addition, the study designs were not uniform. The inherent limitations of non-randomized studies in meta-analysis may have influenced our results. More prospective-design RCTs are needed. As the immune cell types used, combined therapies, patient location, and tumor metastasis status are quite different, especially in the different countries, the evaluation criteria of outcomes can be diverse. These problems are solved with the random-effects method and subgroup analysis. The meta-analysis is also limited by the literature quality. Several studies had inconsistent endpoints and the data were insufficient for conducting further exploration. Potential publication bias cannot be ignored, as most of the studies had been performed in Asia. Due to the relatively new development period, there are no eligible articles with published results related to specific cells such as CAR-T, CAR-NK, and TCR-T cells. Considering the above limitations, our meta-analysis results should be interpreted with caution.

## Conclusions

As a new approach to tumor treatment, cellular immunotherapy has the advantages of good safety, high efficiency, and wide indications. Although widely used in a variety of tumors, its curative effect on liver cancer remains limited. Local infusion is an improved mode for enhancing antineoplastic efficacy and reducing systemic toxicity. We demonstrate that cell therapy through local perfusion is safe and effective for patients with liver cancer. It is more efficacious when combined with minimally invasive interventional therapy. Our findings may help promote the clinical application of cellular immunotherapy in cancer treatment. Given the limitations on the quantity and quality of the included studies, more high-quality prospective clinical trials are warranted to confirm our conclusion. Moreover, how the specific recognition ability of immune cells for liver tumor can be improved and further increase therapeutic efficacy, is also the focus of cellular immunotherapy in the future.

## Data Availability Statement

The original contributions presented in the study are included in the article/**Supplementary Material**. Further inquiries can be directed to the corresponding author.

## Author Contributions

SC and WL conceived and designed the study. SC and HC performed the literature search and data abstraction. SC and YZ were involved in the data analysis and interpretation. SC wrote the initial draft. WL reviewed and edited the manuscript. All authors read and approved the final manuscript.

## Funding

This work was supported by the Capital’s Funds for Health Improvement and Research (CFH 2020-2-2175) and Beijing Talents Project.

## Conflict of Interest

The authors declare that the research was conducted in the absence of any commercial or financial relationships that could be construed as a potential conflict of interest.

## Publisher’s Note

All claims expressed in this article are solely those of the authors and do not necessarily represent those of their affiliated organizations, or those of the publisher, the editors and the reviewers. Any product that may be evaluated in this article, or claim that may be made by its manufacturer, is not guaranteed or endorsed by the publisher.
