# Supplementary material for: Efficacy and Safety of Cellular Immunotherapy by Local Infusion for Liver Tumor: A Systematic Review and Meta-Analysis
Source: Front Oncol. 2022 Feb 28;12:772509. doi: 10.3389/fonc.2022.772509 (PMC8918675; doi:10.3389/fonc.2022.772509)
Supplement: Supplementary file 1 [file DataSheet_1.docx]

Supplementary Material

# Supplementary Methods

**Search strategy**

**Web of Science**

#1 ALL=((lymphokine activated killer) OR (cytokine induced killer) OR (natural killer) OR (dendritic cell) OR (tumor infiltrating lymphocyte) OR (chimeric antigen receptor) OR (T cell receptor) OR LAK OR CIK OR NK OR DC OR TIL OR CAR-T OR CAR-NK OR TCR-T OR immunocytotherapy)

#2 ALL=(local OR regional OR hepatic OR HAI OR TACE OR (intraarterial OR intraartery OR intralesional OR intratumoral OR arterial OR artery))

#3 ALL=(infusion OR perfusion OR transfusion OR implantation OR injection OR refusion OR reinfusion)

#4 ALL=(((hepatocellular OR hepatic OR liver OR hepatocyte*) AND (carcinom* OR cancer* OR neoplas* OR malign* OR tumor* OR metastas*)) OR HCC)

#5 (#4 AND #3 AND #2 AND #1)

**Embase**

#1 ‘liver tumor’/exp OR ‘liver cancer’/exp OR ‘liver cell carcinoma’/exp OR ‘liver metastasis’/exp

#2 (hepatocellular OR hepatic OR liver OR hepatocyte*) AND (carcinom* OR cancer* OR neoplas* OR malign* OR tumor* OR metastas*)

#3 hcc

#4 ‘lymphokine activated killer’ OR ‘cytokine induced killer’ OR ‘natural killer’ OR ‘dendritic cell’ OR ‘tumor infiltrating lymphocyte’ OR ‘chimeric antigen receptor’ OR ‘t cell receptor’ OR lak OR cik OR nk OR dc OR til OR ‘car-t’ OR ‘car-nk’ OR ‘tcr-t’ OR immunocytotherapy

#5 local OR regional OR hepatic OR hai OR tace OR intraarterial OR intraartery OR intralesional OR intratumoral OR arterial OR artery

#6 infusion OR perfusion OR transfusion OR implantation OR injection OR refusion OR reinfusion

#7 (#1 OR #2 OR #3)

#8 (#4 AND #5 AND #6 AND #7)

**Cochrane Library**

#1 MeSH descriptor: [Carcinoma, Hepatocellular] explode all trees

#2 MeSH descriptor: [Liver Neoplasms] explode all trees

#3 ((hepatocellular OR hepatic OR liver OR hepatocyte*) AND (carcinom* OR cancer* OR neoplas* OR malign* OR tumor* OR metastas*)) OR HCC

#4 (#1 OR #2 OR #3)

#5 (lymphokine activated killer) OR (cytokine induced killer) OR (natural killer) OR (dendritic cell) OR (tumor infiltrating lymphocyte) OR (chimeric antigen receptor) OR (T cell receptor) OR LAK OR CIK OR NK OR DC OR TIL OR CAR-T OR CAR-NK OR TCR-T OR immunocytotherapy

#6 local OR regional OR hepatic OR HAI OR TACE OR (intraarterial OR intraartery OR intralesional OR intratumoral OR arterial OR artery)

#7 infusion OR perfusion OR transfusion OR implantation OR injection OR refusion OR reinfusion

#8 (#4 AND #5 AND #6 AND #7)

# Supplementary Figures


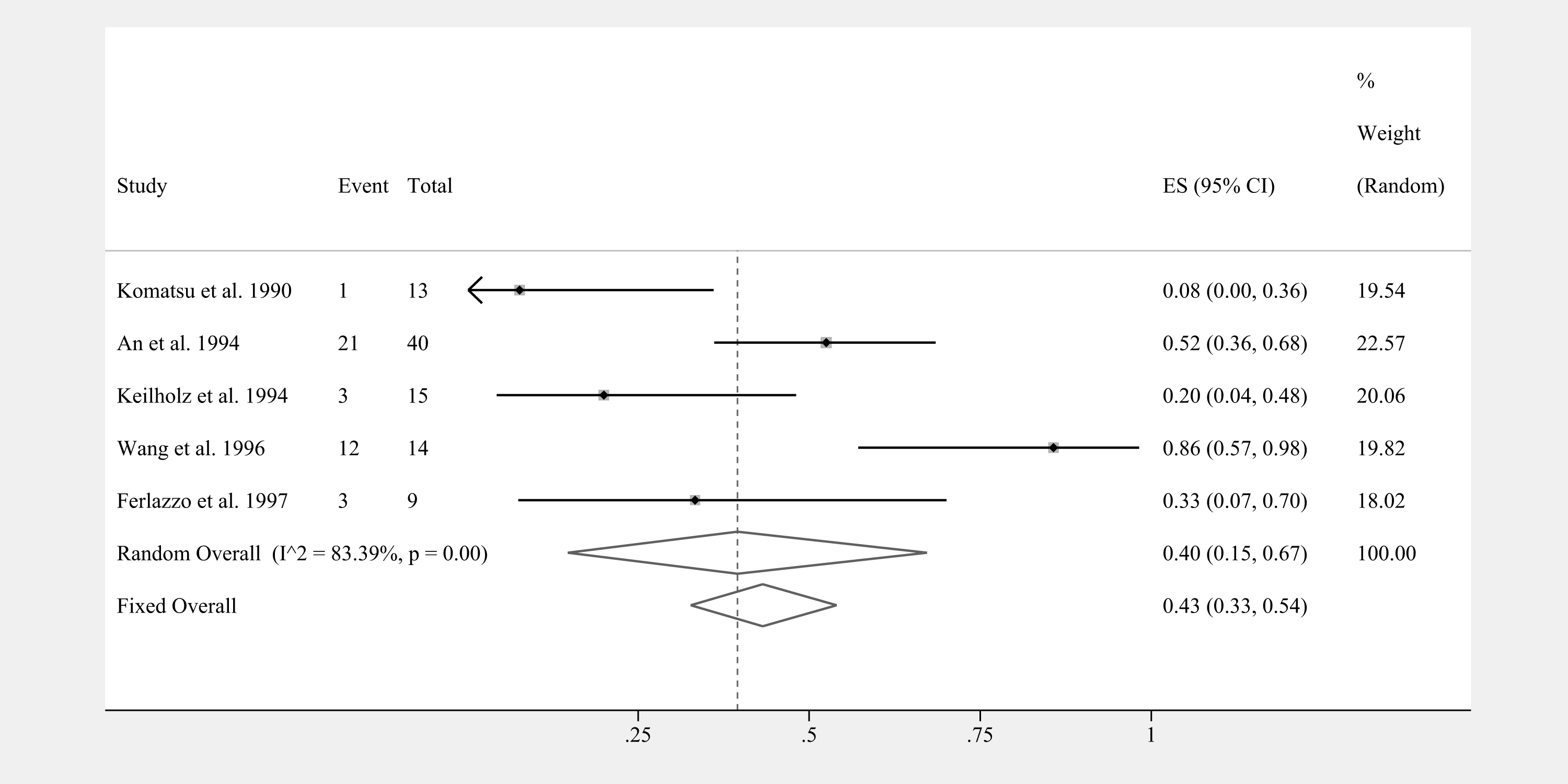


**Supplementary Figure 1.** Forest plots showing pooled analysis of ORR in LAK group.


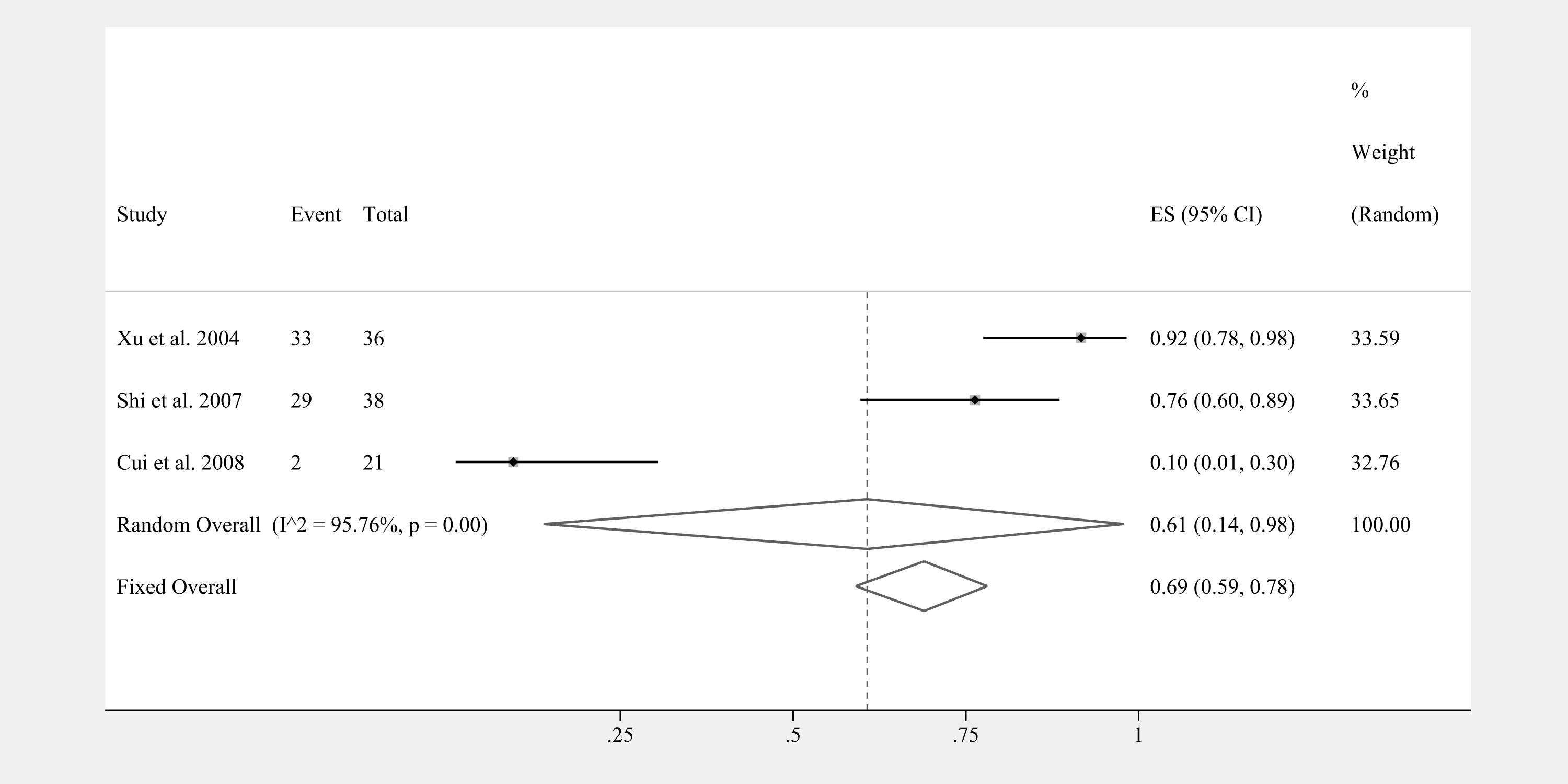


**Supplementary Figure 2.** Forest plots showing pooled analysis of ORR in CIK group.


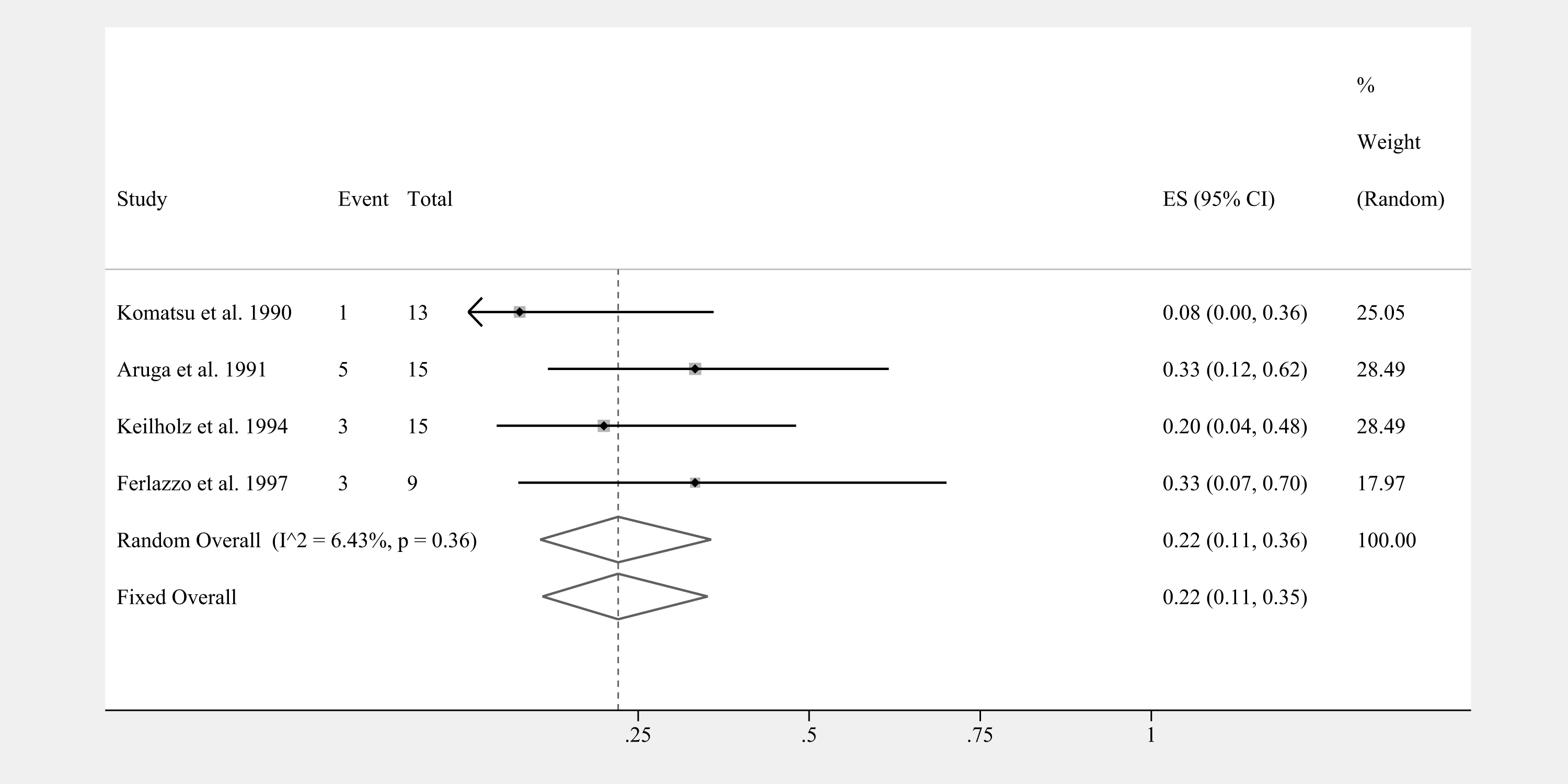


**Supplementary Figure 3.** Forest plots showing pooled analysis of ORR in group using cell therapy alone.


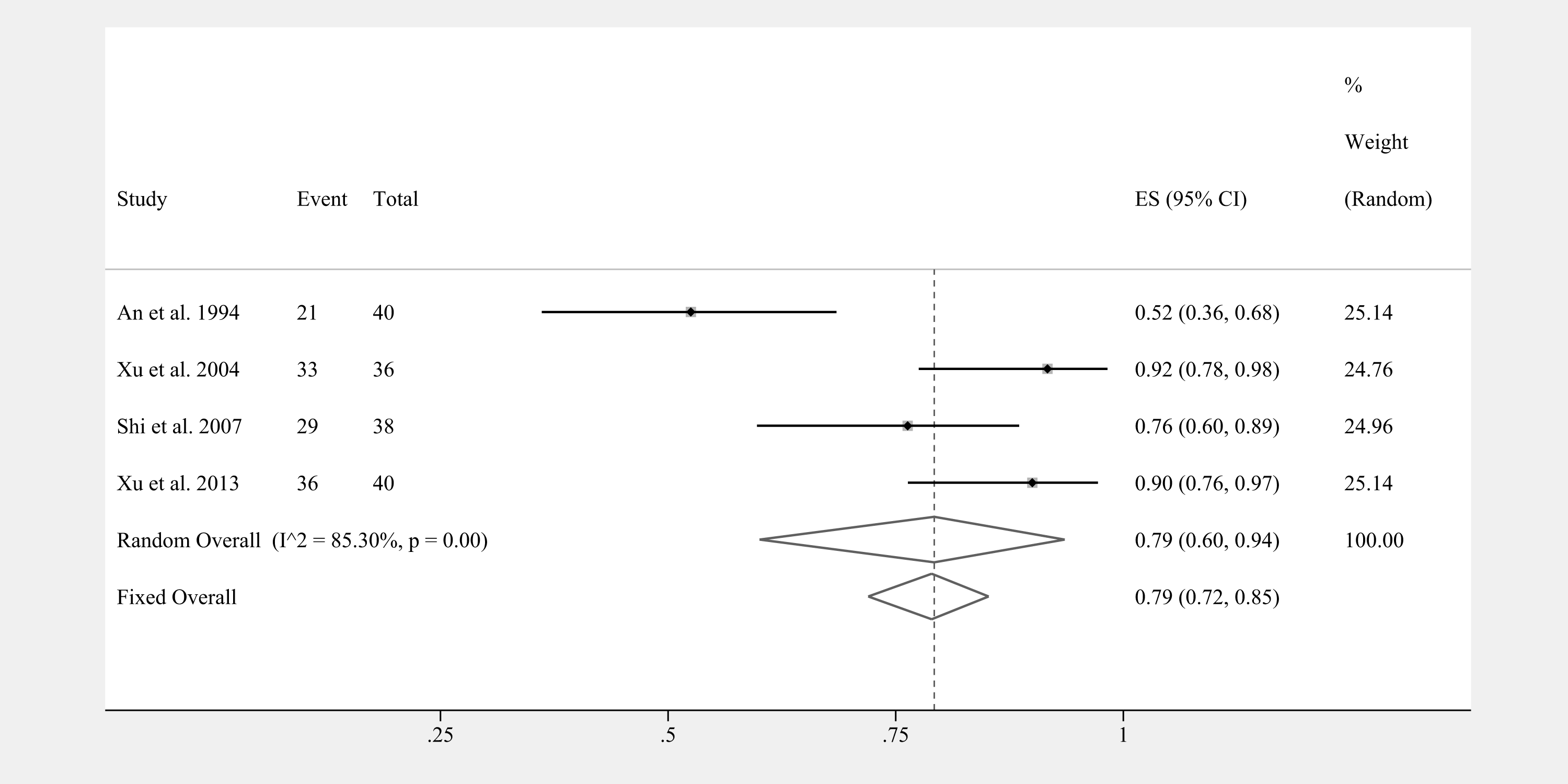


**Supplementary Figure 4.** Forest plots showing pooled analysis of ORR in group using cell therapy combined with minimally invasive treatment.


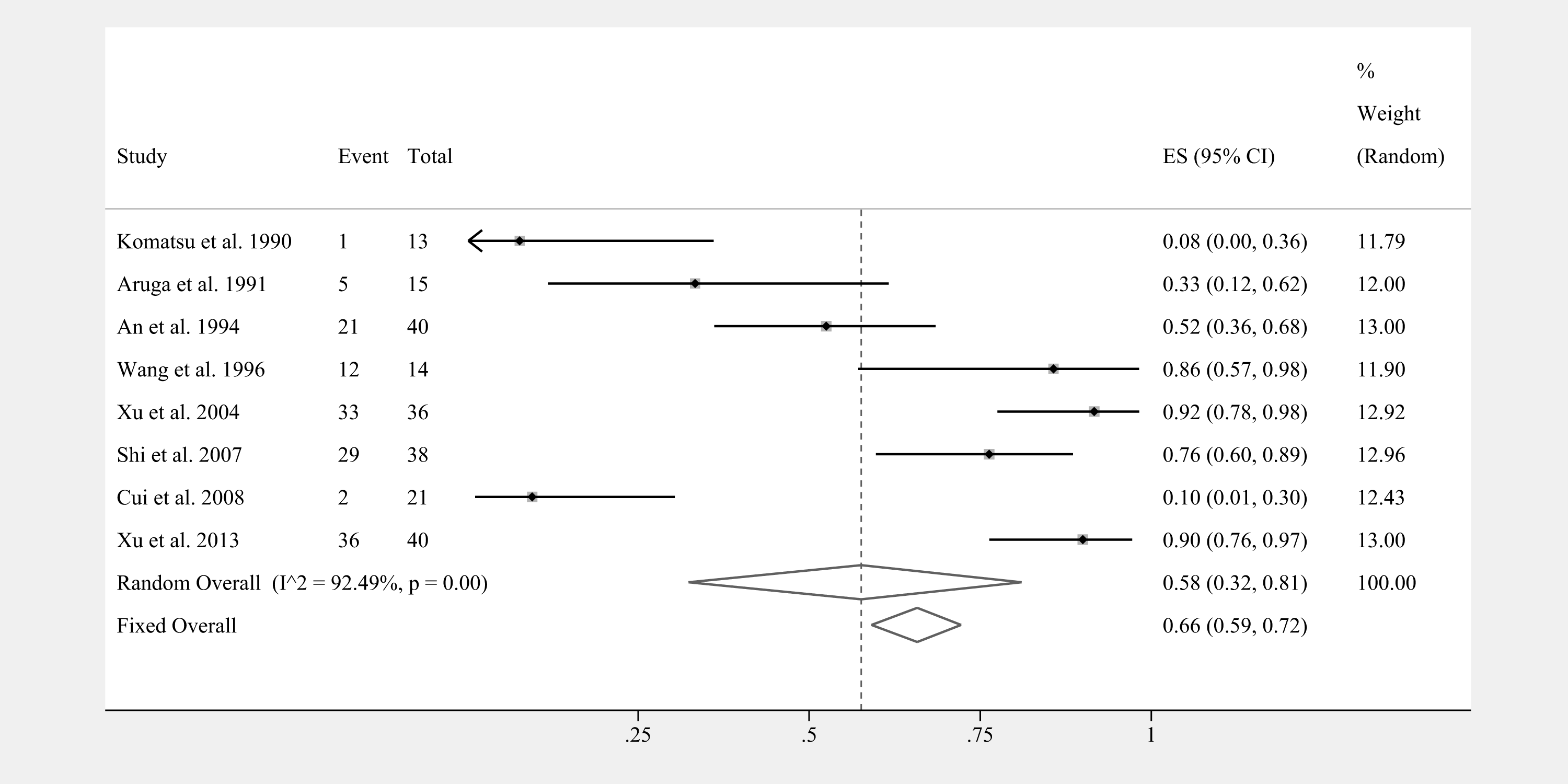


**Supplementary Figure 5.** Forest plots showing pooled analysis of ORR in Asian group.


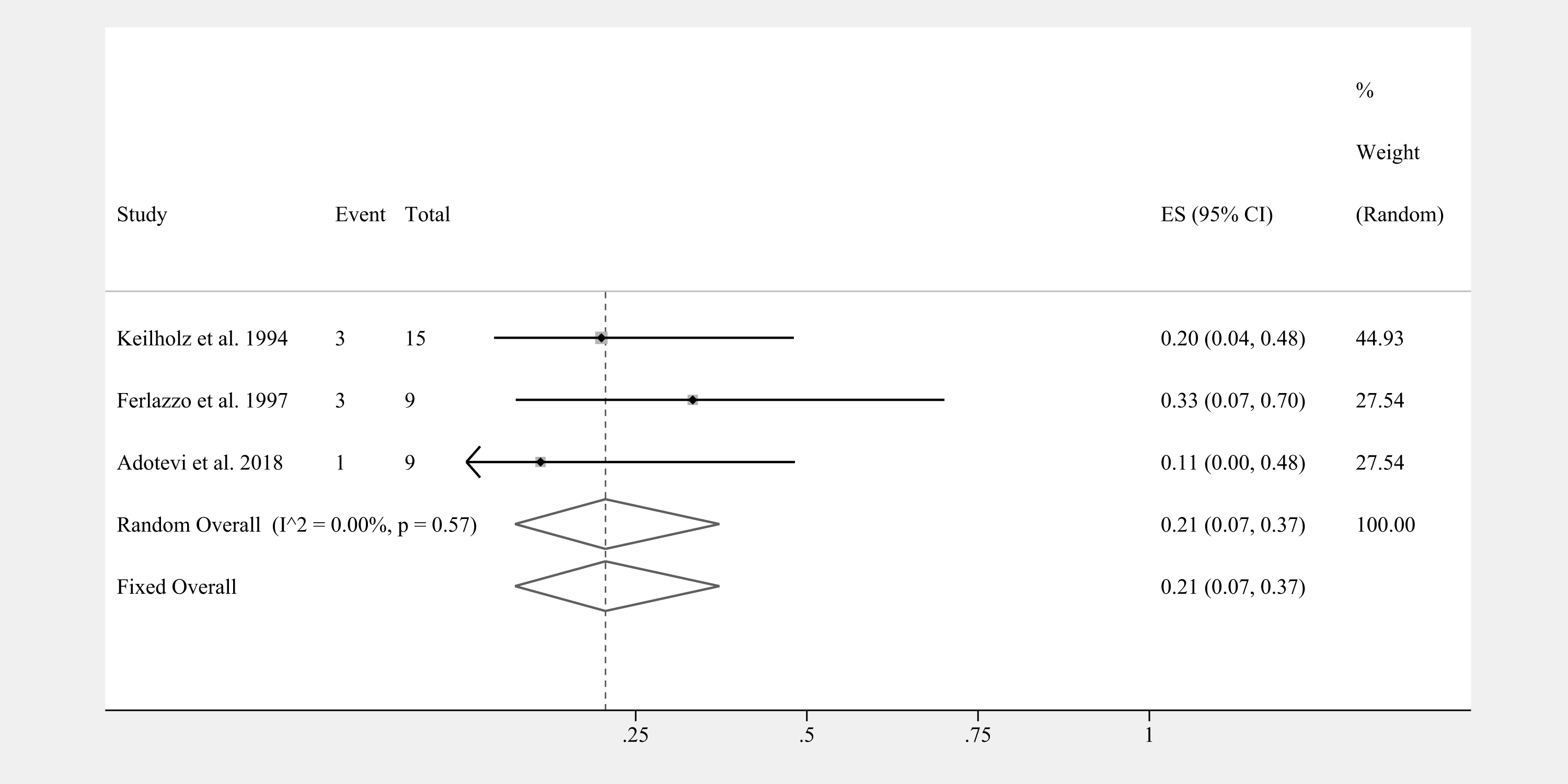


**Supplementary Figure 6.** Forest plots showing pooled analysis of ORR in European group.


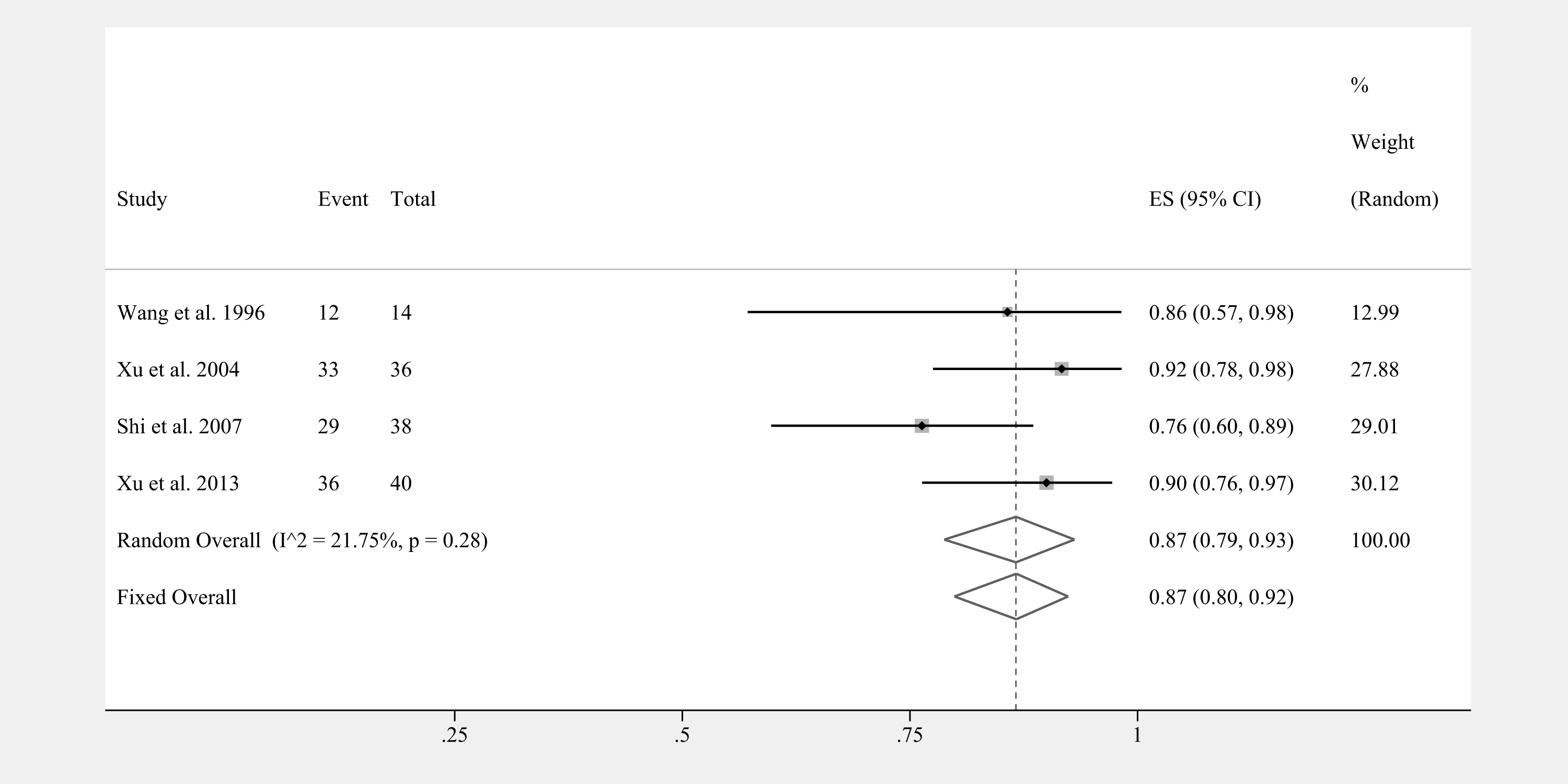


**Supplementary Figure 7.** Forest plots showing pooled analysis of ORR in HCC group.


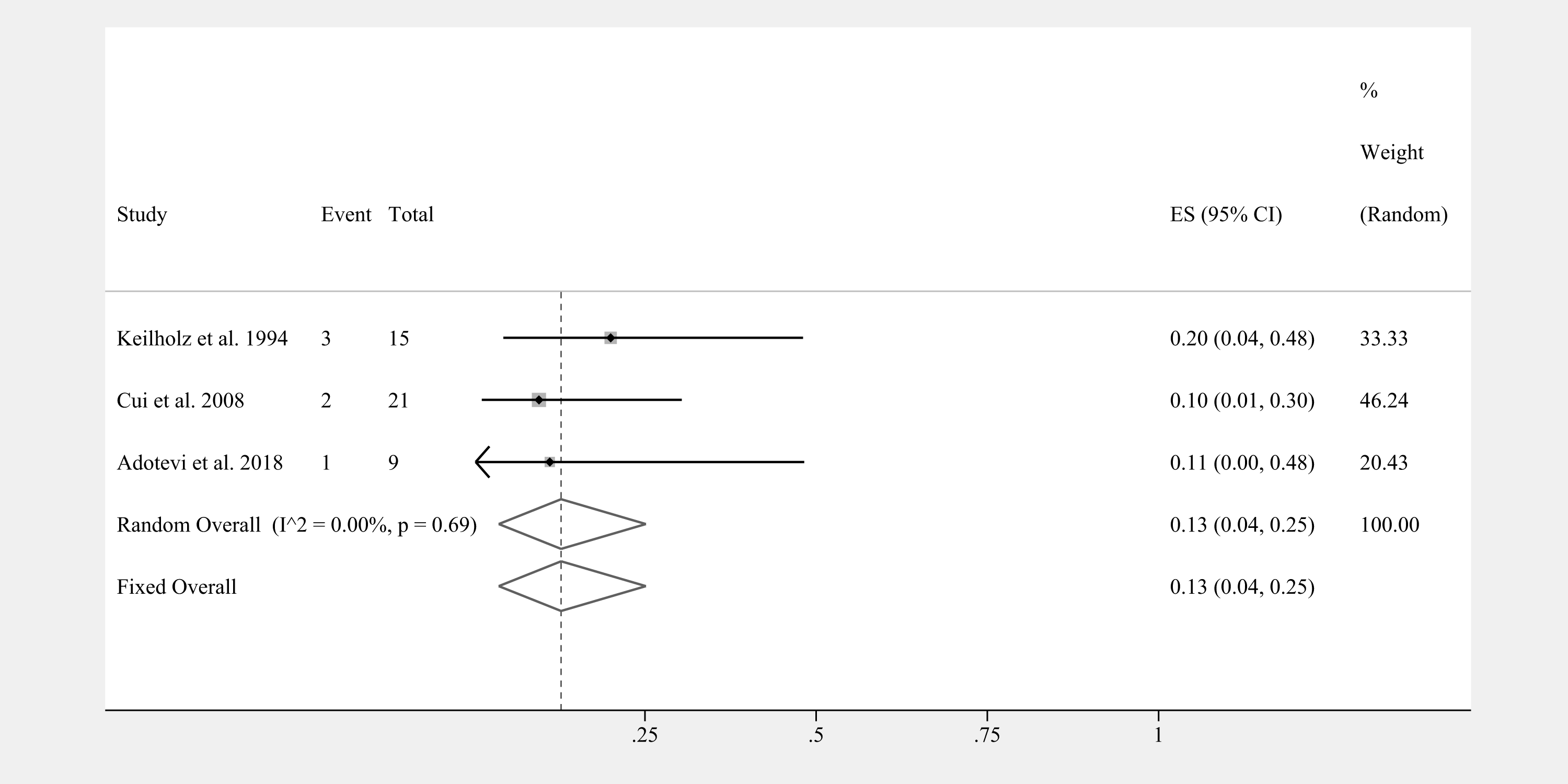


**Supplementary Figure 8.** Forest plots showing pooled analysis of ORR in liver metastases group.


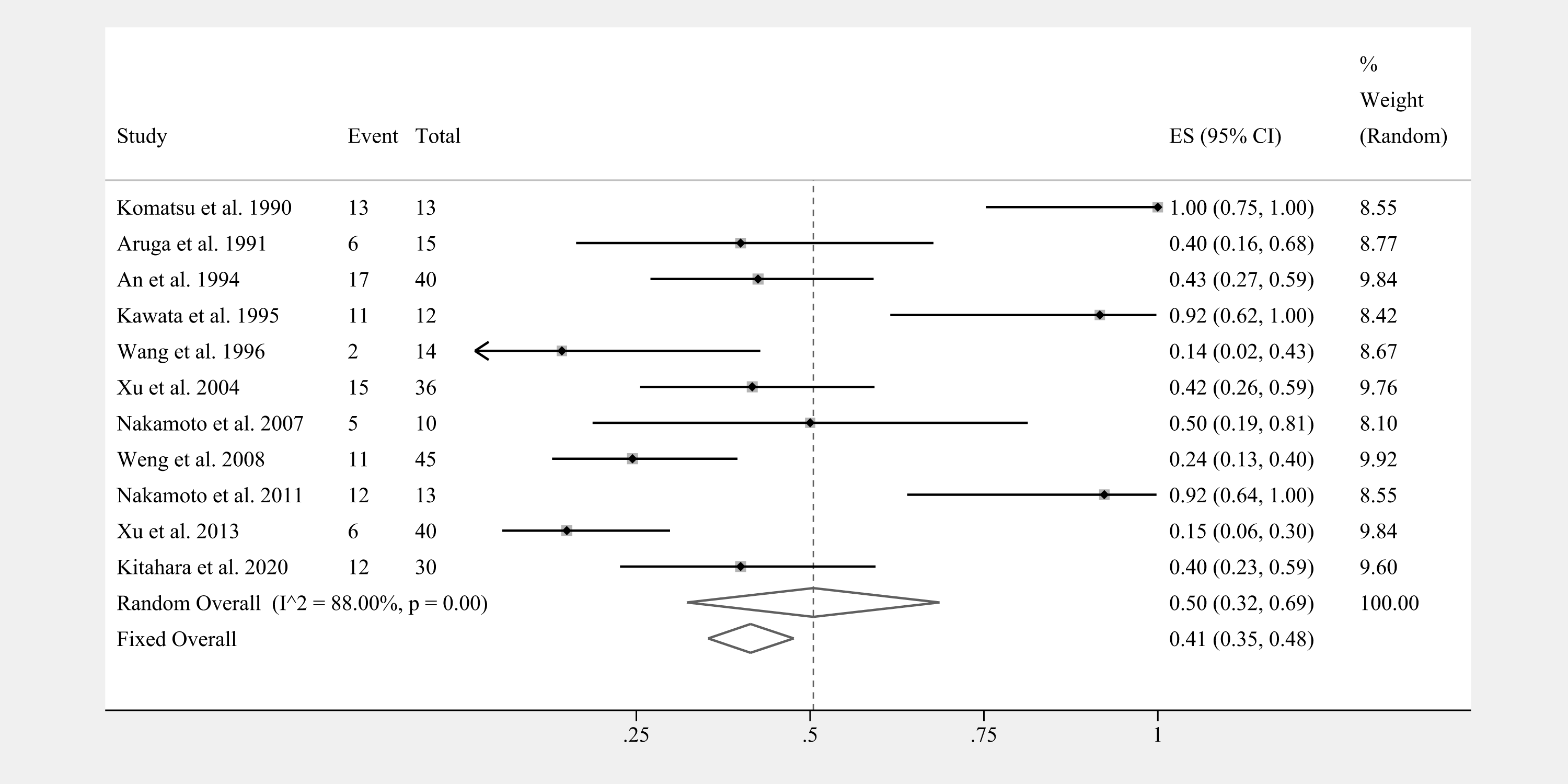


**Supplementary Figure 9.** Forest plots showing pooled analysis on incidence of fever.


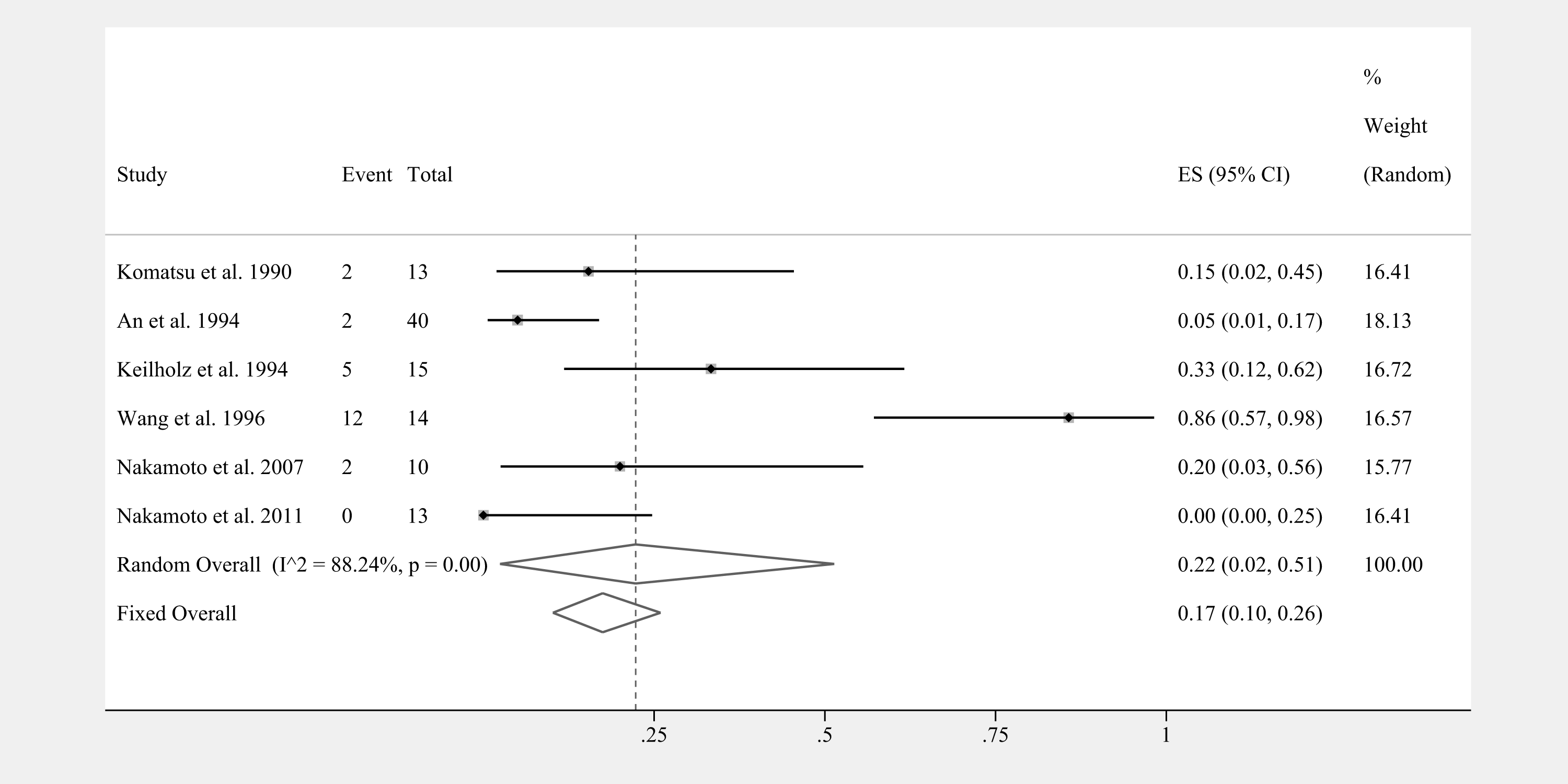


**Supplementary Figure 10.** Forest plots showing pooled analysis on incidence of gastrointestinal toxicity.


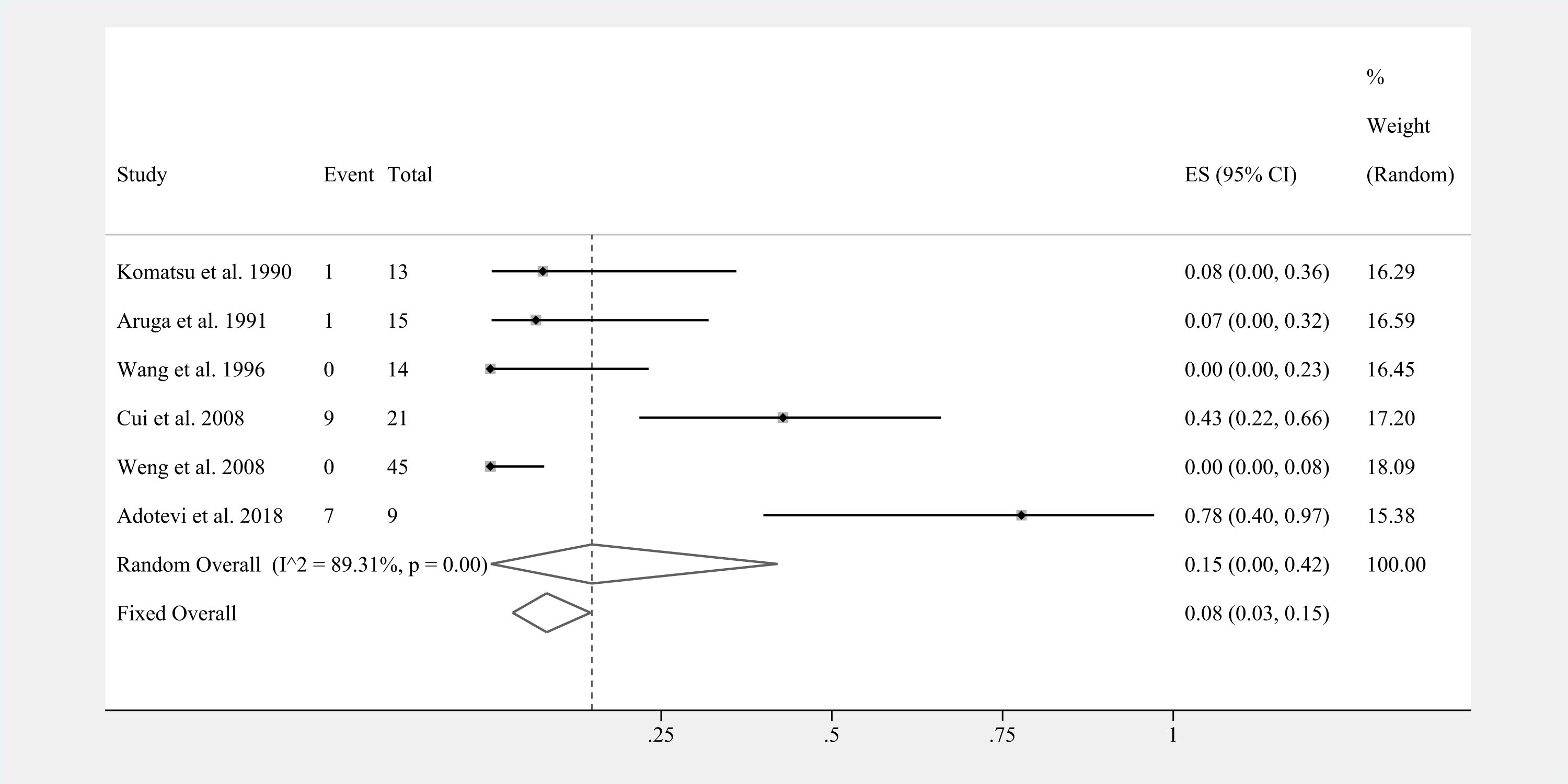


**Supplementary Figure 11.** Forest plots showing pooled analysis on incidence of hepatic dysfunction.


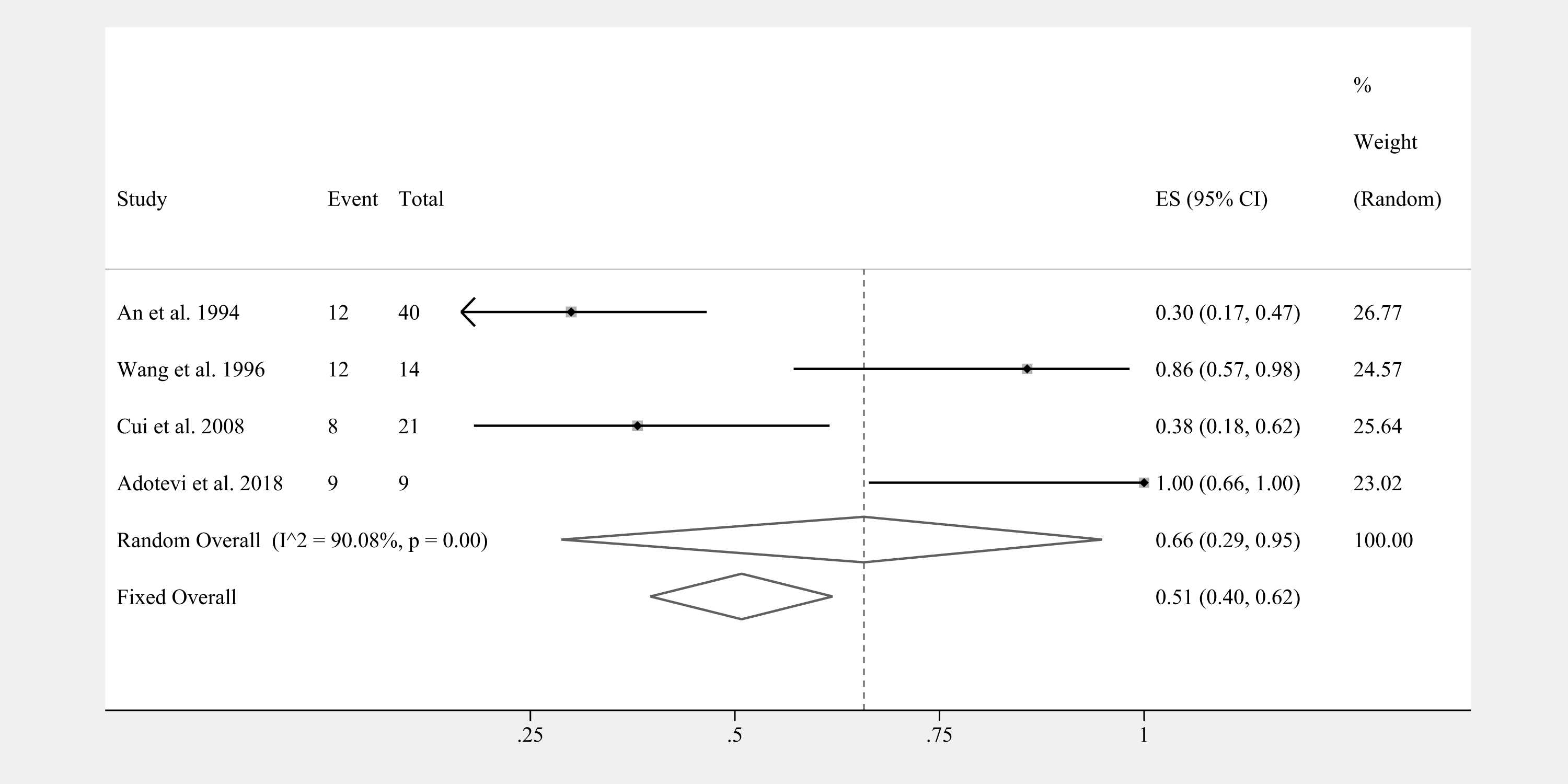


**Supplementary Figure 12.** Forest plots showing pooled analysis on incidence of myelosuppression.


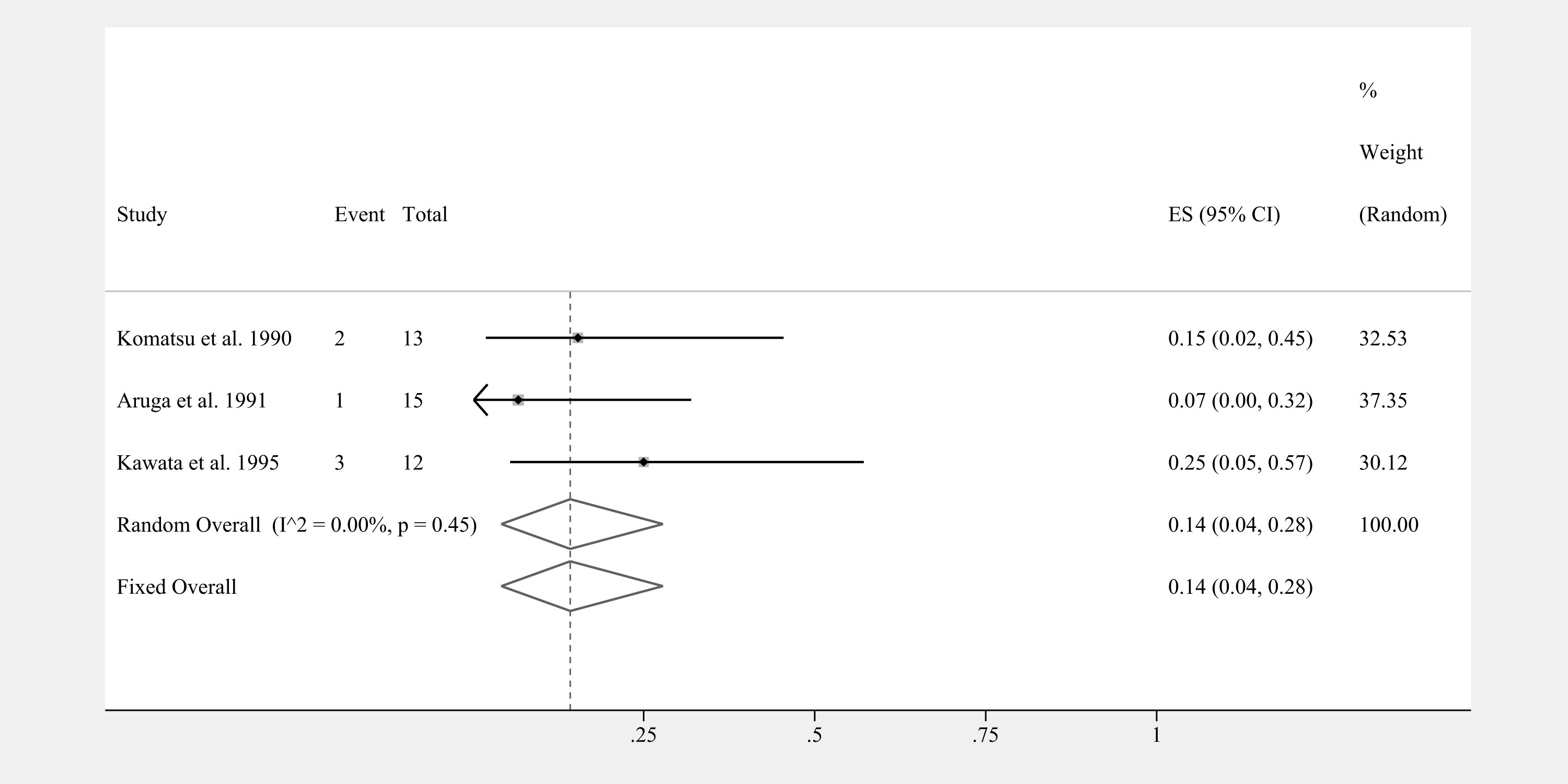


**Supplementary Figure 13.** Forest plots showing pooled analysis on incidence of pleural effusion and/or ascites.


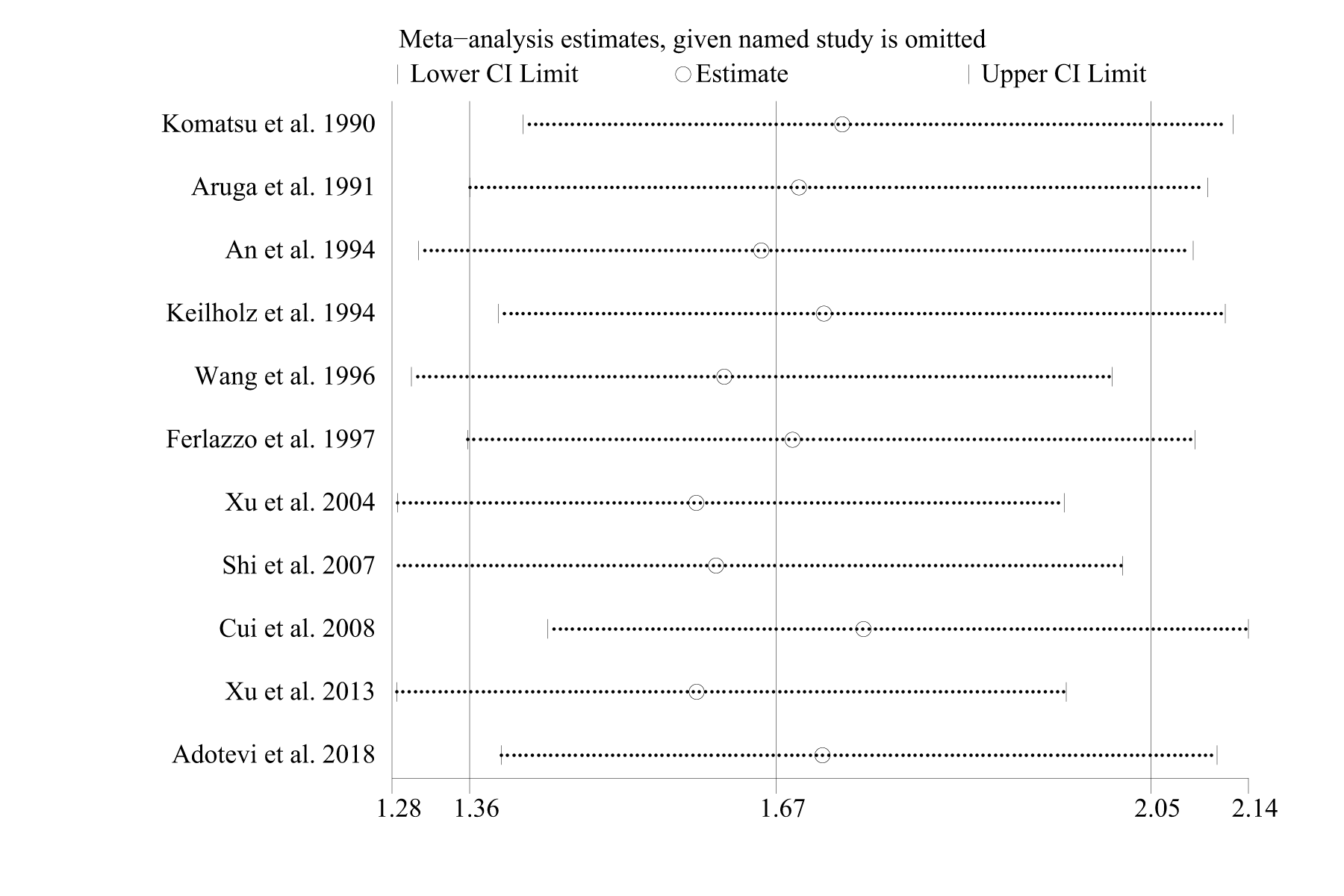


**Supplementary Figure 14.** Sensitivity analysis on the pooled ORR.


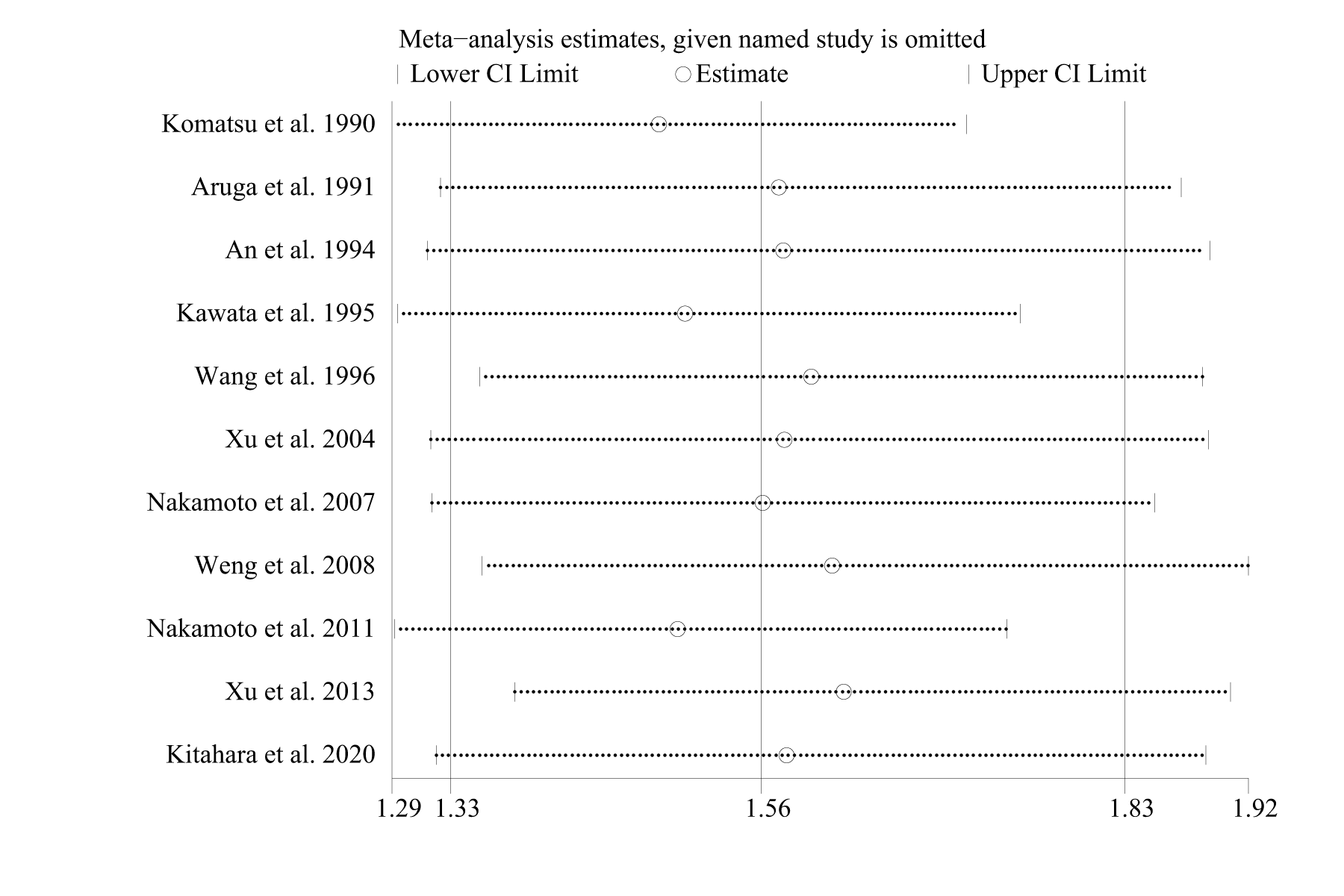


**Supplementary Figure 15.** Sensitivity analysis on the pooled fever rate.
